# Treatment with benznidazole and pentoxifylline regulates microRNA transcriptomic profile in a murine model of Chagas chronic cardiomyopathy

**DOI:** 10.1371/journal.pntd.0011223

**Published:** 2023-03-27

**Authors:** Priscila Silva Grijó Farani, Beatriz Iandra da Silva Ferreira, Khodeza Begum, Glaucia Vilar-Pereira, Isabela Resende Pereira, Edith A. Fernández-Figueroa, Roberto Alejandro Cardenas-Ovando, Igor C. Almeida, Sourav Roy, Joseli Lannes-Vieira, Otacilio Cruz Moreira

**Affiliations:** 1 Real-Time PCR Platform RPT09A, Laboratory of Molecular Virology and Parasitology, Oswaldo Cruz Institute, Oswaldo Cruz Foundation, Rio de Janeiro, Brazil; 2 Laboratory of Biology of the Interactions, Oswaldo Cruz Institute, Oswaldo Cruz Foundation, Rio de Janeiro, Brazil; 3 Border Biomedical Research Center, Department of Biological Sciences, University of Texas at El Paso, El Paso, Texas, United States of America; 4 Computational and Integrative Genomics, Instituto Nacional de Medicina Genómica, Arenal Tepepan, Mexico City, Mexico; McGill university, CANADA

## Abstract

Chronic Chagas cardiomyopathy (CCC) is one of the leading causes of morbidity and mortality due to cardiovascular disorders in endemic areas of Chagas disease (CD), a neglected tropical illness caused by the protozoan parasite *Trypanosoma cruzi*. CCC is characterized by parasite persistence and inflammatory response in the heart tissue, which occur parallel to microRNA (miRNA) alterations. Here, we investigated the miRNA transcriptome profiling in the cardiac tissue of chronically *T*. *cruzi*-infected mice treated with a suboptimal dose of benznidazole (Bz), the immunomodulator pentoxifylline alone (PTX), or the combination of both (Bz+PTX), following the CCC onset. At 150 days post-infection, Bz, PTX, and Bz+PTX treatment regimens improved electrocardiographic alterations, reducing the percentage of mice afflicted by sinus arrhythmia and second-degree atrioventricular block (AVB2) when compared with the vehicle-treated animals. miRNA Transcriptome profiling revealed considerable changes in the differential expression of miRNAs in the Bz and Bz+PTX treatment groups compared with the control (infected, vehicle-treated) group. The latter showed pathways related to organismal abnormalities, cellular development, skeletal muscle development, cardiac enlargement, and fibrosis, likely associated with CCC. Bz-Treated mice exhibited 68 differentially expressed miRNAs related to signaling pathways like cell cycle, cell death and survival, tissue morphology, and connective tissue function. Finally, the Bz+PTX-treated group revealed 58 differentially expressed miRNAs associated with key signaling pathways related to cellular growth and proliferation, tissue development, cardiac fibrosis, damage, and necrosis/cell death. The *T*. *cruzi*-induced upregulation of miR-146b-5p, previously shown in acutely infected mice and *in vitro T*. *cruzi*-infected cardiomyocytes, was reversed upon Bz and Bz+PTX treatment regimens when further experimentally validated. Our results further our understanding of molecular pathways related to CCC progression and evaluation of treatment response. Moreover, the differentially expressed miRNAs may serve as drug targets, associated molecular therapy, or biomarkers of treatment outcomes.

## Author summary

Chronic Chagas cardiomyopathy (CCC) is a neglected tropical illness caused by the *Trypanosoma cruzi*, characterized a persistent inflammatory response driven by parasite persistence in the infected tissues. In this study, authors investigated the miRNA expression pattern in the cardiac tissue of chronically *T*. *cruzi*-infected mice treated with benznidazole (Bz), the immunomodulator pentoxifylline alone (PTX), or the combination of both (Bz+PTX). All treatment regimens improved electrocardiographic alterations and clinical parameters, while miRNA profiling revealed considerable alterations in expression. Infected group showed pathways related to organismal abnormalities, cellular development, skeletal muscle development, cardiac enlargement, and fibrosis, likely associated with CCC. Bz group is mainly affecting cell cycle, cell death and survival, tissue morphology, and connective tissue function pathways. On the other hand, Bz+PTX-treated group is mainly affecting cellular growth and proliferation, tissue development, cardiac fibrosis, damage, and necrosis/cell death pathways. Surprisingly, miR-146b-5p upregulation in the infected group was reversed upon Bz and Bz+PTX treatment regimens, highlighting its importance as a biomarker of therapeutic response. Our results help understanding the molecular pathways related to CCC progression and evaluation of treatment response in Chagas disease.

## Introduction

Chagas disease (CD), also known as American trypanosomiasis, is caused by the infection of the protozoan parasite *Trypanosoma cruzi*, which can infect a wide range of sylvatic and domestic mammalians and can be transmitted by more than 150 triatomine vectors [1]. It is estimated that 6–7 million people are infected, and approximately 75 million are exposed to the risk of infection [2]. Chagas disease has been considered endemic to rural regions of Latin America, where vector transmission is the main route of infection [3,4], causing a loss of approximately 750,000 working days due to premature death and $1.2 billion in productivity losses yearly [5]. Additionally, since human migration has considerably increased in the last several years, the infection has been disseminated to countries not originally endemic, such as the United States, Spain, Japan, and Australia. The lack of screening in blood banks and the inexperience of health professionals in diagnosing and managing the disease has contributed to organ transplant and congenital transmissions [1,6], causing an economic burden of $7 billion/year for the global public health [4,7].

Chagas disease has an acute and a chronic phase. The acute phase comprises a 4-8-week duration, with patent parasitemia and inflammation due to tissue parasitism, generally asymptomatic or unspecific symptoms [8,9]. Trypanocidal therapy is effective if administered during the acute phase, with a cure rate ranging from 60–85% [10]. Nevertheless, the main problem relies on adequately diagnosing and treating the disease in that phase, which, if not done, may progress to the chronic phase, where the parasitemia is mostly undetectable [9]. Most patients progress to the indeterminate form of CD. They may never show clinical signs of the disease during their lifespan, mostly diagnosed by serological and/or parasitological positivity but lacking electrocardiographic (ECG) and/or radiological abnormalities [9]. On the other hand, 30–40% of patients progress to the determinate forms of chronic CD (CCD), which may be cardiac, digestive, or cardiodigestive [11]. The cardiac form (CCC) of CCD is the most frequent and severe form of the disease, characterized by an intense and intermittent inflammatory response due to parasite persistence [12], being one of the leading causes of morbidity and mortality due to cardiovascular disorders in endemic areas [13]. It is proposed that CCC originates from a dysregulation of the immune response triggering a robust cardiac remodeling process causing hypertrophy, fibrosis, and edema, consequently affecting the electrophysiological properties of the heart leading to life-threatening arrhythmias that may cause late manifestations such as thromboembolism, heart failure, and sudden death [1,8,9].

Presently, benznidazole (Bz) and nifurtimox (Nf) are the only two drugs approved for CD treatment. Both have been used consistently for the last 50 years, although their efficacy and safety rates could be better due to their high rate of adverse events [3]. Bz belongs to the group of nitroheterocyclic compounds, being a prodrug that exerts its effect after activation by the trypanosomal type-I nitroreductase, intrinsic of *T*. *cruzi* and other protozoa, thus producing reactive metabolites that have a trypanocidal effect on the intra- and extracellular forms of the parasite [10,14], being more commonly used than Nf due to its better tolerability and safety [3]. However, due to its relatively high rate of adverse events, which caused ~13% permanent treatment discontinuation in the BENEFIT clinical trial [15], special attention has been given to establishing an etiological treatment using lower doses of Bz. This has already been demonstrated in an *in vitro* and *in vivo* model to have the same trypanocidal efficacy as the standard dose [16]. Therefore, recent clinical trials have focused on new therapeutic regimens to optimize the existing pharmacotherapy without changing its effectiveness, using lower doses or lower dose frequency of Bz for shorter or extended treatment periods. Among them, BENDITA [17,18], MULTIBENZ [19], and TESEO [20] studies have already been concluded or are in progress [21]. In this regard, the importance of optimizing pharmacotherapy in the CD treatment and, especially in the treatment of CCC, is evident. In the present study, we also give this focus and use one-quarter of the standard Bz dose in the treatment of the murine CCD model.

Pentoxifylline (PTX) is a methylxanthine derivative traditionally used to treat peripheral vascular diseases that also has anti-inflammatory and cardioprotective properties [22]. Among PTX immunomodulatory properties, it is shown to decrease the production of critical proinflammatory cytokines such as TNF, IL-1, and IL-6, which consequently affect the activity of monocytes and macrophages, favoring a Th2-type cytokine induction while decreasing the Th1-type inflammatory response [23]. Moreover, PTX has immunomodulatory effects independent of its TNF modulation action, such as inhibition of the proliferation of peripheral mononuclear cells, decreased adhesion to the cellular matrix, and decreased IL-12 production [24]. Previously, PTX has been explored by our group as a complementary immunomodulatory therapy in the treatment of CCC in chronically *T*. *cruzi*-infected C57BL/6 mice, where PTX promoted reduction of CD8^+^ T cells expressing markers of activation and migration in the spleen and also the activation of cardiac blood vessel endothelial cells, in addition to reducing myocarditis, cardiac tissue damage progression and ameliorating ECG parameters [25]. Moreover, we also evaluated the combined Bz+PTX treatment in the same experimental model, showing a reduction in heart tissue parasite load, inflammation, and fibrosis, promoting improvement in ECG alterations caused by *T*. *cruzi* infection. Additionally, the combined Bz+PTX therapy reduced TNF expression and inducible nitric oxide synthase (iNOS/NOS2) in the cardiac tissue and TNF receptor-1 expression on CD8^+^ T cells [26].

MicroRNAs (miRNAs) are a class of small, single-stranded non-coding RNAs that have 19 to 22 nucleotides and act by preventing transcription or inducing degradation of the target mRNA, being generally involved in inhibiting gene expression at the post-transcriptional level [27,28], as they are the most abundant class of gene regulatory molecules in animals [29]. miRNAs play a crucial role in regulating gene expression of cardiac signaling pathways during proliferation, differentiation, metabolism, apoptosis, angiogenesis, and pathological processes [30]. They are also involved in establishing several pathological processes related to viral, bacterial, and parasite infections [31–34]. *T*. *cruzi* infection has already been shown to alter host miRNA expression levels in the cardiac tissue of patients with CCC [35,36] and in murine models [37–39]. Evaluation of the expression of nine miRNAs in the cardiac tissue of patients with CCC revealed that five miRNAs (miR-1, miR-133a, miR-133b, miR-208a, and miR-208b) were down-regulated in patients with CCC when compared with healthy individuals and patients with idiopathic dilated cardiomyopathy (DCM) [35]. Additionally, miRNA transcriptome profiling done in cardiac tissue of acutely *T*. *cruzi*-infected mice identified nine miRNAs (miR-21, miR-142-5p, miR-142-3p, rno-miR-146b, mmu-miR-146b, miR-222, miR-145, miR-322, and miR-149) showed significant correlation with parasitemia and the prolongation of the corrected QT (QTc) interval in the ECG. Furthermore, the prediction of miRNA targets revealed key molecules such as gap junction protein alpha 5 (GJA5) and potassium voltage-gated channel subfamily A member 1 (KCNA1), both closely related to changes in the electrical conduction of the heart, especially the QTc interval [38]. A more recent study on miRNAs in CD, investigated the molecular mechanisms of miRNAs and mRNAs differentially expressed in myocardial tissue of CCC patients, showing that differentially expressed miRNAs (DEMs) were involved in processes related to CCC onset, including fibrosis, hypertrophy, myocarditis, and arrhythmias. Pathway analysis with targets for the DEMs revealed their involvement in immune response and metabolism, in which IFN-γ, TNF, and NF-κB played a central role [35,36]. Moreover, analysis of signaling pathways showed activation of inflammation-related pathways such as Th1 response, IFN-γ-induced genes, fibrosis, hypertrophy, and mitochondrial/oxidative and antioxidant stress responses [36].

Thus far, no other study has investigated the expression of miRNAs in a murine model of CCC undergoing new etiological therapy regimens aiming to maintain the Bz efficacy while decreasing its adverse events, similar to what has been done in recently completed or ongoing clinical trials. Therefore, here we investigate the miRNA transcriptome profiles in the cardiac tissue of *T*. *cruzi*-infected mice under a suboptimal Bz dose or combined Bz+PTX treatment. This study aims to unveil the main molecular pathways affected by the parasite infection and therapeutic regimens, thus establishing possible targets for the DEMs and contributing to the elucidation of new therapeutic biomarkers or complementary therapy candidates.

## Material and methods

### Ethical statements

This study was carried out in strict accordance with recommendations in the Guide for the Care and Use of Laboratory Animals of the Brazilian National Council of Animal Experimentation (https://www.mctic.gov.br/mctic/opencms/institucional/concea) and the Federal Law 11.794 (8 October 2008). The Institutional Committee for Animal Ethics of Fiocruz (CEUA-Fiocruz L004/09 and LW10/14) approved all experimental procedures used in the present study. All presented data were obtained from two independent experiments (Experiment Register Book #49, #53, and #57, LBI/IOC-Fiocruz).

### Experimental *T*. *cruzi* infection and drug treatment

Mice were obtained from the animal facilities of the Oswaldo Cruz Foundation (ICTB/Fiocruz, Rio de Janeiro, Brazil). Immediately after arrival, mice were housed in polypropylene cages under specific-pathogen-free conditions, randomly grouped into three mice per cage. Cages were maintained in microisolators under standard conditions (with temperature and relative humidity of ~22°C ± 2°C and 55% ± 10%, respectively), noise and light (12-h light-dark cycle) control, and mice received grain-based chaw food and water *ad libitum*. To minimize stress, mice were kept in adaptation for 10–14 days in a plastic igloo-enriched cage. After adaptation, five to 7-week-old female C57BL/6 (H-2^b^) mice were intraperitoneally (i.p.) infected with 100 blood-derived trypomastigotes (BDTs) of *T*. *cruzi* Colombian strain (DTU TcI) in 0.2 ml of vaccine-grade sterile buffered saline (BioManguinhos/Fiocruz, Brazil). After 120 days post-infection (dpi), animals in the chronic phase presenting clinical signs of CCC [26,40,41] received an intraperitoneal (i.p.) injection with apyrogenic saline (noninfected, vehicle-treated group) or saline containing PTX (Trental, Sanofi-Aventis) at 20 mg/Kg and/or one-quarter of Bz optimal dose (¼ Bz; 25 mg/Kg/day; LAFEPE) by gavage using apyrogenic water (BioManguinhos/Fiocruz, Brazil), daily, for 30 days. Cardiac alterations were monitored by ECG, before (120 dpi) and after (150 dpi) therapy. At 150 dpi, mice were euthanized under anesthesia (ketamine 300 mg/Kg + xylazine 30 mg/Kg). Hearts were collected in RNA*later* Stabilization Solution (Invitrogen) for processing before performing the molecular assays.

### ECG registers

Mice were tranquilized with diazepam (10 mg/Kg), and transducers were placed subcutaneously (DII). The traces were recorded for 2 min using a digital Power Lab 2/20system connected to a bio-amplifier at 2mV for 1 second (PanLab Instruments, Spain). The filters were standardized between 0.1 and 100 Hz, and the traces were analyzed using Scope software for Windows V3.6.10 (PanLab Instruments, Spain). The ECG parameters were analyzed as previously described [40].

### DNA extraction and *T*. *cruzi* parasite load quantification by quantitative real-time PCR

Mice were randomly selected for each group. Genomic DNA was extracted from 10–20 mg of mouse hearts using High Pure PCR Template Preparation Kit (Roche Diagnostics, Indianapolis, IN), following the manufacturer”s instructions. Before extraction, tissues were withdrawn from RNA later and disrupted in 500 μL of tissue lysis buffer from the High Pure PCR Template Preparation Kit (Roche), using a TissueRuptor II (QIAGEN, USA) at its maximum speed for 30 sec. The ensuing homogenate was submitted to DNA extraction using the kit above, following the manufacturer”s recommendations. At the last step of the protocol, DNA was eluted from the silica column in 100 μL of elution buffer and stored at– 20°C until further analysis. Amplification of *T*. *cruzi* satellite DNA was done by using the specific primers Cruzi1 (5′–ASTCGGCTGATCGTTTTCGA–3′) and Cruzi2 (5′–AATTCCTCCAAGCAGCGGATA–3′), both at 750 nM and the TaqMan probe Cruzi3 (6FAM–CACACACTGGACACCAA–NFQ–MGB) at 50 nM with 10 μL FastStart Universal Probe Master Mix 2X (Roche Diagnostics GmbHCorp. Mannheim, Germany) in a final volume of 20 μL. As an endogenous internal control, the predesigned TaqMan assay targeting mouse GAPDH gene (Cat n°. Mm99999915-g1, Applied Biosystems) was used [42]. Standard curves were done by spiking 1 x 10^6^ BDTs (Colombian strain), obtained from infected VERO cells, into 30 mg heart tissue of a noninfected mouse. Following DNA extraction, a 1:10 serial dilution of the eluted DNA in ultrapure water (from 10^6^ to 0.1 parasite equivalents) was done. Real-time PCR reactions were performed on an Applied Biosystems ViiA 7 Real-Time PCR (Thermo Fisher Scientific, USA) thermocycler, using the following cycling conditions: 50°C for 2 min, 94°C for 10 min, followed by 40 cycles at 95°C and 58°C for 1 min, where sample fluorescence was acquired after each cycle. All samples were run in duplicate, and a threshold was set at 0.02 for both targets.

### Total RNA extraction

10–20 mg of mouse heart tissues were withdrawn from RNA later and disrupted in 500 μL lysis buffer using TissueRuptor II (QIAGEN, USA) at maximal speed for 30 sec. Total RNA was extracted using mirVana miRNA Isolation Kit (Life Technologies), according to the manufacturer”s recommendations. Total RNA quantification and purity were assessed in a NanoDrop ND2000 (Thermo Fisher), and integrity was analyzed in a Bioanalyzer 2100 (Agilent, USA) using RNA Nano 6000 kit. Only samples with RIN ≥ 7.5 were used in this study.

### MicroRNA expression profiling by quantitative real-time PCR

A pool of three total RNA samples, extracted from cardiac tissue of noninfected mice and infected-treated (vehicle, Bz, or Bz+PTX) mice were used for the gene expression analysis of 752 miRNAs and four reference miRNAs candidates, according to the Applied Biosystems protocols. Reverse transcription was performed from 1,000 ng total RNA using Megaplex RT Primers (A+B), Rodent Pool (Applied Biosystems, Thermo Fisher Scientific, Cat no. 4444746) with TaqMan MicroRNA Reverse Transcription Kit (Applied Biosystems, Thermo Fisher Scientific, Cat no. 4366596). The multiplexed RT reaction was performed according to manufacturer”s instructions. Quantitative real time RT-qPCR was done utilizing pre-printed TLDA 384 wells microfluidic cards (TaqMan Array Rodent MicroRNA A+B Cards Set v3.0, Applied Biosystems, Thermo Fisher Scientific, Cat no. 4444909) that contained FAM/NFQ-MGB labelled probes specific to mature miRNAs and 4 endogenous small nucleolar RNAs candidates for data normalization and relative quantification. The reaction mix was performed with 450 μL TaqMan Universal PCR Master Mix 2X, 6 μL Megaplex RT product (sample), and 444 μL nuclease-free water. Furthermore, 100 μL sample+master mix was loaded onto each microfluidic channel, centrifuged twice at 1,200 rpm for 1 min and mechanically sealed with the Applied Biosystems sealer device, following manufacturer”s instructions. Real-time PCR reactions were carried out on Applied Biosystems ViiA 7 Real-Time PCR (Thermo Fisher) thermocycler, using the cycling conditions: 10 min at 95°C, followed by 40 cycles of 15 sec at 95°C and 60 sec at 60°C. Fluorescence was collected after each cycle, at the annealing/extension step. Raw data files were pre-processed using QuantStudio Real-Time PCR Software (Applied Biosystems) with threshold and baseline corrections for each sample and gene expression results were analyzed and Expression Suite v1.0.3 (Applied Biosystems). Threshold was set at 0.08 for all miRNAs. After the stability score analysis of the reference small RNA candidates, using the Expression Suite Software, U87 and snoRNA135 were selected as the most-stable reference gene pair (score = 0.274). Gene expression was estimated by the ΔΔCt method [43,44] with global normalization, using the noninfected group as calibrator.

### Pathway analysis

QIAGEN”s Ingenuity Pathway Analysis (IPA) software [45] (IPA, Qiagen, Redwood City, CA) was used to examine the direct and indirect relationships between the miRNAs and their potential targets within the CCC-related pathways. IPA is a web-based application for data analysis in pathway context. Lists of DEMs and their respective fold change values were uploaded and used as input for the QIAGEN IPA. MiRNAs were mapped to the IPA knowledgebase using the miRNA IDs. The miRNA target filter analysis was used to find the targets for the respective mRNAs. The targets that have been experimentally observed previously and those predicted at high-confidence levels were selected. Core analysis was performed with the putative targets to identify canonical pathways and top diseases and functions. The default maximum and minimum values set by IPA were used. The “Grow” tool was used to visualize the direct/indirect relationships among the molecules within the pathways and the miRNA dataset. The “Grow” tool only shows the connections between miRNAs and mRNAs (miRNA-mRNA); therefore, the “Connect” tool was also used to visualize more direct/indirect connections, those between miRNA-miRNA and mRNA-mRNA with a previously chosen confidence level. The figures were finalized using the “Path Designer” tool, which provides more clarity in differentiating the connections among the different types of molecules, and were downloaded directly from the IPA network visualization tool.

### Analysis of individual gene expression by RT-qPCR

Reverse transcription reactions of the mature microRNAs: U87 (assay ID 001712), miR-145-5p (assay ID 002278), and miR-146b-5p (assay ID 001097) were performed with 10 ng of total RNA using TaqMan MicroRNA Reverse Transcription Kit (Applied Biosystems, Thermo Fisher Scientific, USA/Cat no. 4366596) and their respective stem-loop primers, following manufacturer”s instructions. RT reactions (15 μL) were run in an Eppendorf Mastercycler thermocycler at 16°C for 30 min, 42°C for 30 min, and 85°C for 5 min. Quantitative real-time RT-qPCR was done in a 10 μL reaction containing 5 μL of 2x TaqMan Universal PCR Master Mix, 0.5 μL of TaqMan probe belonging to either U87, miR-145-5p or miR-146b-5p, 2 μL of cDNA and 2.5 μL of RNase-free water. Real-time PCRs were carried out on Applied Biosystems ViiA 7 Real-Time PCR (Thermo Fisher, USA) thermocycler, using the cycling conditions: 10 minutes at 95°C, followed by 40 cycles of 15 seconds at 95°C and 60 seconds at 60°C. Fluorescence was collected after each cycle at the annealing/extension step. Raw data files were pre-processed using QuantStudio Real-Time PCR Software (Applied Biosystems, USA) with baseline corrections when necessary. Gene expression results were estimated with Expression Suite v1.0.3 (Applied Biosystems, USA) with a threshold set at 0.02 for all targets. U87 was used as the reference miRNA once it had down constitutive expression across samples. Target miRNA levels were estimated by the ΔΔCt method [43,44], using noninfected samples as calibrators.

### Statistical analysis

All experiments were performed in at least three technical replicates. To assess the normality of the data, the Shapiro-Wilk test was used. To determine whether there were any significant statistical differences between the groups, unpaired one-way ANOVA with Tukey`s multiple comparisons with a 95% confidence level was used. Correlation analysis was done using Pearson”s correlation coefficient. All statistical tests were performed using GraphPad Prism 9.0 (GraphPad Software, San Diego, CA). The data were expressed as mean plus standard deviation (SD), and differences were considered statistically significant when p < 0.05.

## Results

### A suboptimal dose of Bz and combined Bz+PTX treatments reversed relevant ECG abnormalities in the chronic Chagas cardiomyopathy experimental model

**Fig 1:**
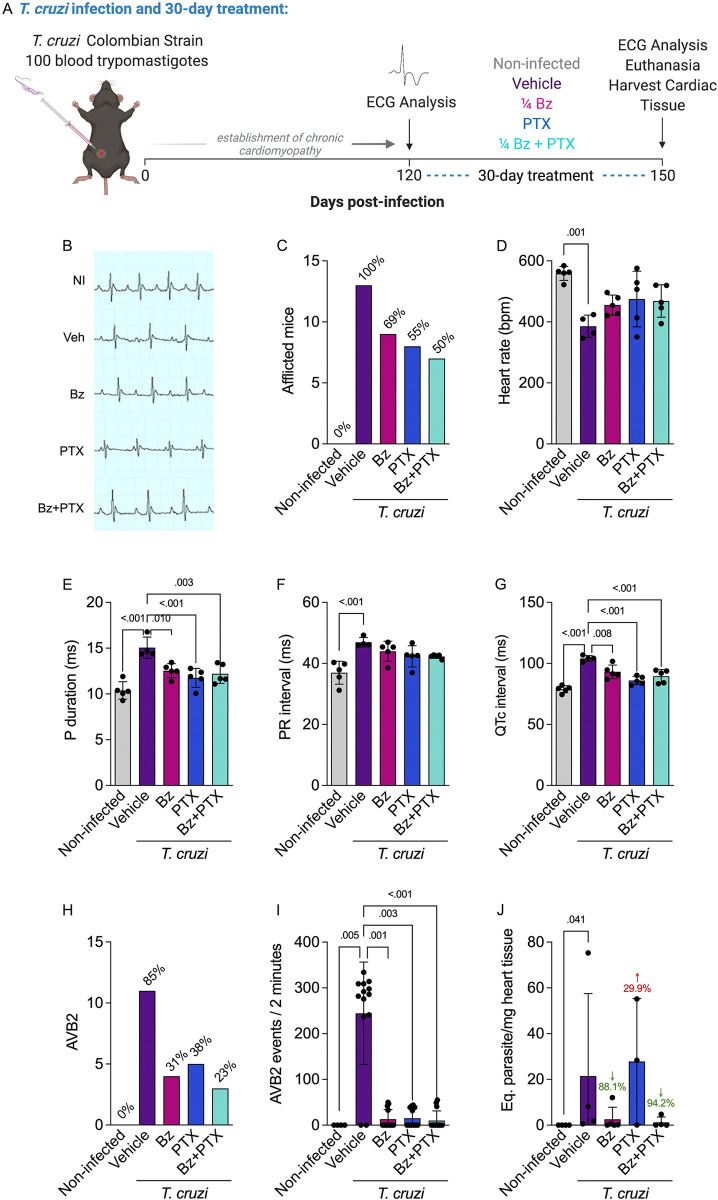
Chronic Chagas cardiomyopathy model and treatment with a suboptimal dose of Bz, PTX, or Bz+PTX. (A) Experimental design of infection and treatment. Mice were infected and treated daily from 120 to 150 dpi with vehicle alone, suboptimal dose of Bz (¼ dose, 25 mg/kg), PTX (20 mg/kg), or a combined therapy of Bz+PTX, and analyzed at 150 dpi. Created with Biorender. (B) Representative ECG register segments of sex- and age-matched noninfected controls and infected mice (150 dpi) injected with vehicle, Bz, PTX or Bz+PTX. (C) Frequency of afflicted mice by any ECG alteration. (D) Frequency of mice afflicted by sinus arrhythmias. (E) Frequency of mice afflicted with a second-degree atrioventricular block (AVB2). (F) Number of AVB2 events in two-minute records. (G) Parasite load based on qPCR detection of *T*. *cruzi* satellite DNA on mice heart tissue. For all graphs, significance was determined using unpaired one-way ANOVA with Tukey’s multiple comparisons with a 95% confidence level.

To evaluate treatment efficacy on *T*. *cruzi* infection, C57BL/6 mice were infected (i.p.) with 100 BDTs (Colombian strain). At 120 dpi, when electrical abnormalities and heart injury were already installed, as previously established in the same experimental model [40], animals were treated with vehicle (i.p.-injected sterile saline and water by gavage), Bz (25 mg/Kg/day), PTX (20 mg/Kg/day), or combined Bz+PTX, for 30 consecutive days (Fig 1A). At 150 dpi, when compared with age-matched noninfected controls, vehicle-injected chronically infected C57BL/6 mice presented substantial ECG alterations including P wave, PR, and QTc interval prolongation, compared with the noninfected group (Fig 1B). Conversely, Bz-, PTX- or Bz+PTX-treated mice exhibited improvement in ECG alterations, when compared with vehicle-treated mice (Fig 1B). All *T*. *cruzi*-infected mice showed ECG abnormalities, decreasing progressively under therapy with Bz (69%), PTX (55%), or Bz+PTX (50%) (Fig 1C). Additionally, vehicle-treated mice showed bradycardia (385.8 ± 36.73 bpm; p = 0.001) when compared with the noninfected group (558.8 ± 22.72 bpm), indicating a tendency to reversion under Bz (455.4 ± 32.77 bpm), PTX (474.9 ± 91.23 bpm), or Bz+PTX (468.6 ± 53.14 bpm) therapy with no statistical significance when compared with the vehicle group (Fig 1D). Among the most common ECG abnormalities, an increased P wave duration was observed in the vehicle-treated group (15.06 ±1.164 ms; p<0.001) when compared with the noninfected group (10.38 ± 0.9559 ms). However, Bz (12.54 ± 0.7646 ms; p = 0.010), PTX (11.76 ± 1.040 ms; p<0.001), or Bz+PTX (12.20 ± 1.046 ms; p = 0.003) therapy was able to reverse this alteration when compared with the vehicle-treated group (Fig 1E). Moreover, an augmented PR interval was observed in the vehicle-treated group (46.94 ± 1.562 ms; p>0.001) when compared with the noninfected group (36.97 ± 3.737 ms). This alteration was not reversed under Bz (44.00 ± 3.270 ms), PTX (42.31 ± 3.491 ms), or Bz+PTX (42.29 ± 0.6409 ms) therapy (Fig 1F). The QTc interval, considered a relevant predictor of outcome in immune dysregulated myocardial impairment and mortality risk factor for CD [46,47] was significantly increased in the vehicle-treated group (104.1 ± 2.418 ms; p<0.001) when compared with the noninfected group (78.84 ± 2.908 ms). This alteration was reversed by Bz (93.11 ± 5.531 ms; p = 0.008), PTX (86.02 ± 3.381 ms; p<0.001), or Bz+PTX (89.55 ± 5.367 ms; p<0.001) therapy (Fig 1G). Second-degree atrioventricular block (AVB2) was present in most (85%) mice from the vehicle-treated group. All three therapies were able to considerably reduce (Bz [2.7 fold], PTX [2.2 fold], and Bz+PTX [3.7 fold]) the percentage of affected mice compared with the vehicle control group (Fig 1H). Additionally, the number of AVB2 events in two-minute records was significantly increased in the vehicle-treated group (244.5 ± 111.8; p = 0.005) when compared with the noninfected group (0.000 ± 0.000), and this ECG abnormality was abrogated in Bz (13.23 ± 20.99; p = 0.001), PTX (15.08 ± 20.02; p = 0.003), or Bz+PTX (10.36 ± 20.76 p<0.001) treated-mice (Fig 1I). Regarding parasite load in the heart, vehicle-treated mice (21.45 ± 36.05 parasite equivalent (par. eq.)/mg heart tissue; p = 0.041) exhibited a significant increase compared with the noninfected group. Bz therapy showed a tendency to reduce parasite burden (2.564 ± 5.315 eq. par/mg tissue; 88% reduction). While PTX treatment did not affect parasite load (27.87 ± 27.53 eq. par/mg tissue; 30% increase), it did not interfere with Bz efficacy, since the combined Bz+PTX therapy also reduced the parasite load (1.255 ± 2.335 eq. par/mg tissue; 94% reduction) (Fig 1J). These results demonstrate that a suboptimal dose of Bz and the combined Bz+PTX therapy could reverse relevant ECG abnormalities in mice infected with *T*. *cruzi* and efficiently control heart parasitism.

### The expression pattern of microRNAs related to key signaling pathways is regulated during chronic Chagas cardiomyopathy

**Fig 2:**
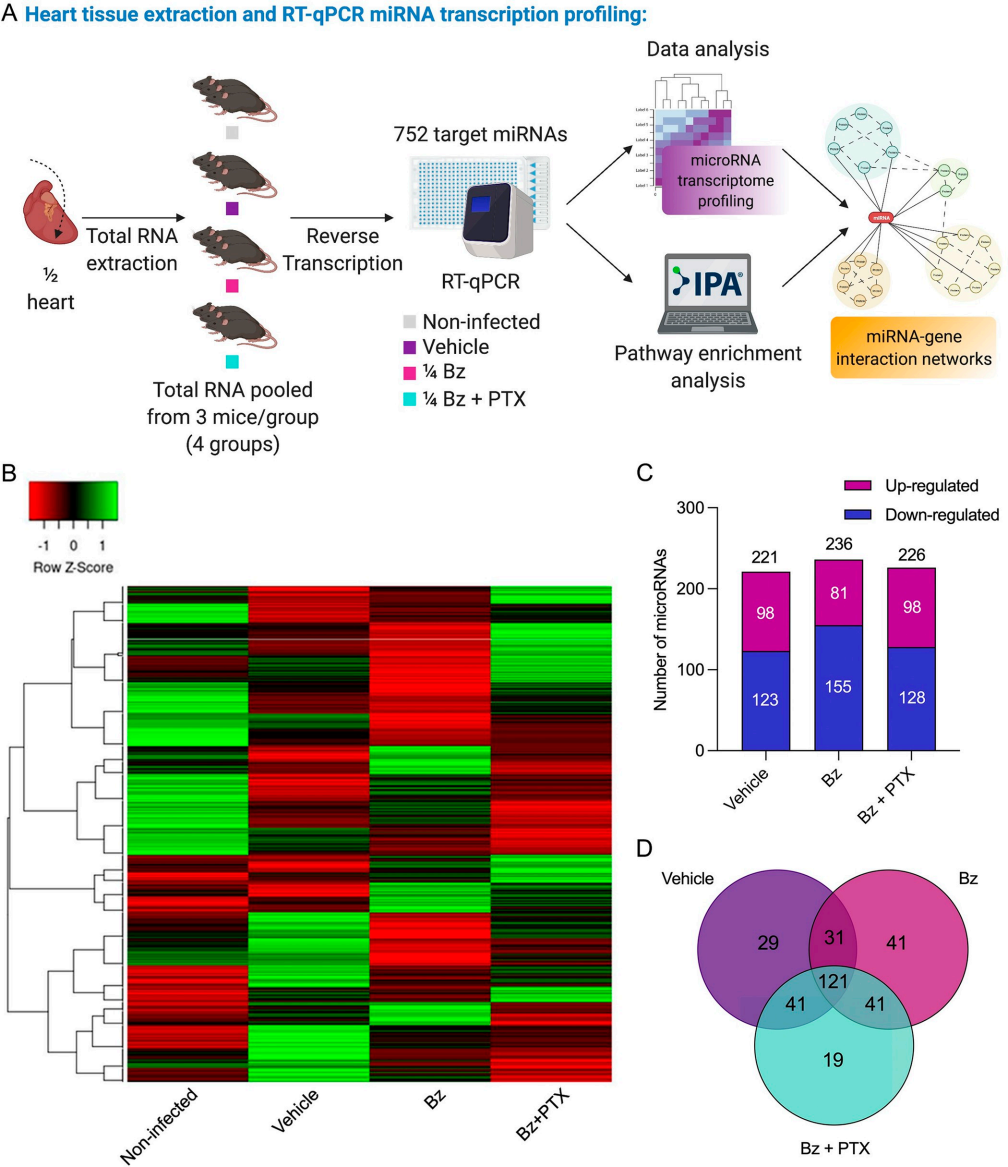
Overview and characterization of immunoarray analysis. (A) Schematic design of array workflow. Murine cardiac tissues were harvested 30 days after therapy and half of the heart was subjected to total RNA extraction, reverse transcription, and RT-qPCR of 752 miRNAs. Created with Biorender. (B) Heatmap and hierarchical clustering. The color scale illustrates the gene fold change after global normalization (row Z-score). Clustering was performed using the average linkage distance measurement method: Spearman Rank correlation. (C) Number of altered genes in each group. (D) Venn diagram showing the number of altered expressed genes in each group.

Next, we analyzed the miRNA expression patterns in the noninfected untreated control group and parasite-infected treated (vehicle, Bz, and Bz+PTX) groups using a TaqMan microRNA array workflow to assess 752 miRNAs in total RNA of mouse cardiac tissue by RT-qPCR (Fig 2A). Out of 752 miRNAs, 370 exhibited amplifications in all experimental groups. Hierarchical clustering revealed that miRNA expression profiles for each target clustered independently between groups, especially the noninfected and vehicle-treated groups that showed almost opposite miRNA expression patterns (Fig 2B). The miRNAs altered by at least 1.5-fold change were selected, revealing a differential miRNA expression for the vehicle-treated group revealed 221 DEMs (98 up and 123 downregulated). In comparison, therapy with Bz showed 236 DEMs (81 up and 155 downregulated), and combined Bz+PTX therapy revealed 226 DEMs (98 up and 128 downregulated) (S1 Table and Fig 2C). Overall, a high overlap differential expression for 222 miRNAs was observed in the three groups analyzed, with 121 DEMs (54.5%) being shared between the groups. Additionally, 29 DEMs were found to be vehicle-specific, 41 DEMs were Bz-specific, and 19 DEMs were Bz+PTX-specific (Fig 2D).

**Fig 3:**
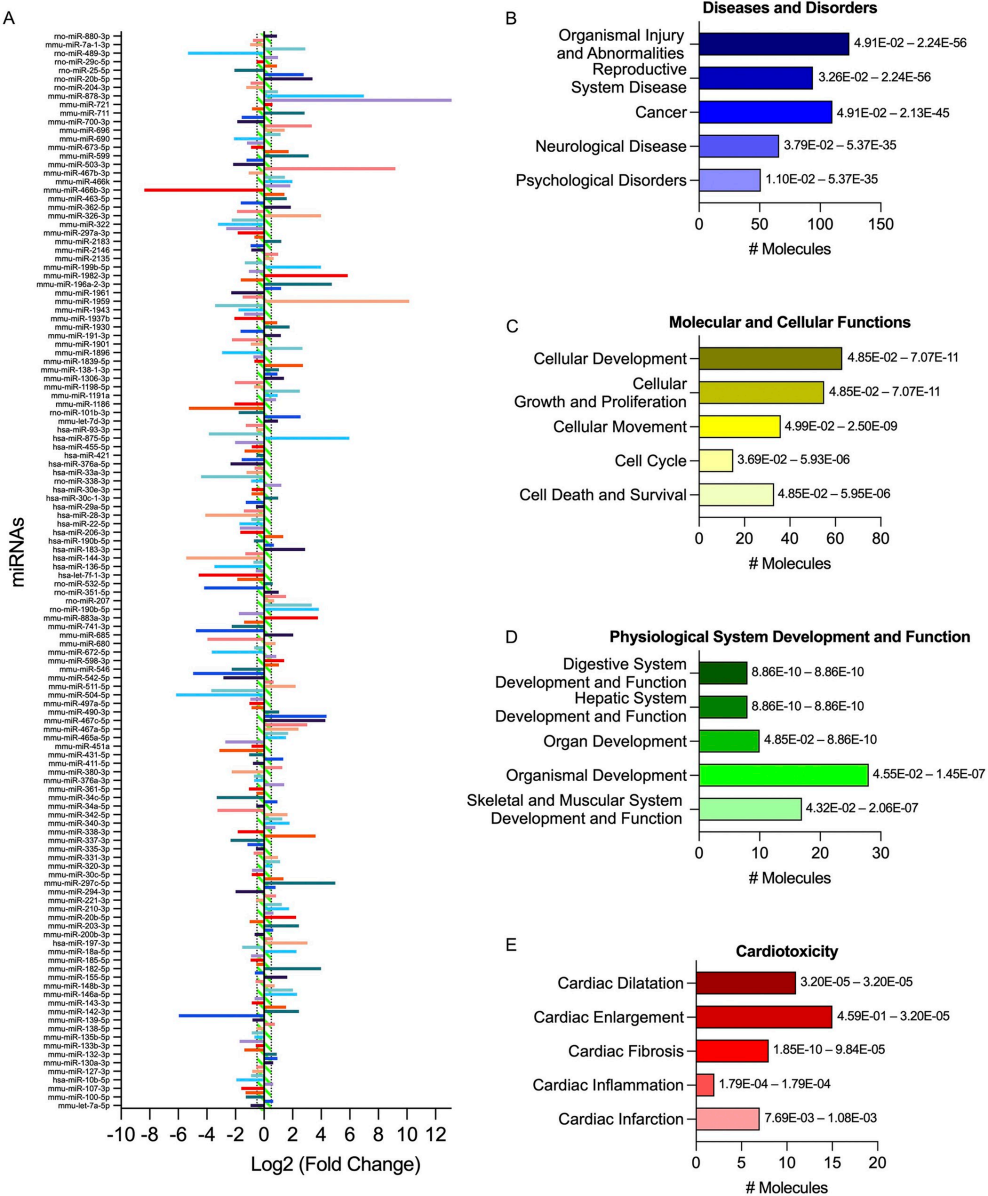
Characterization of the immune response expression profile for the chronically infected group. (A) Overall expression of the altered miRNAs. Results were expressed as log2 fold change, and genes with values greater than 0.5 or lower than -0.5 (1.5-fold change) (dotted vertical lines; green-striped area) were considered altered and used for further analysis on IPA software. (B) Diseases and disorders pathway analysis. (C) Molecular and cellular function pathway analysis. (D) Physiological system development and function pathway analysis. (E) Cardiotoxicity pathway analysis. Significance intervals of enrichment are shown on the right of the bars.

**Fig 4:**
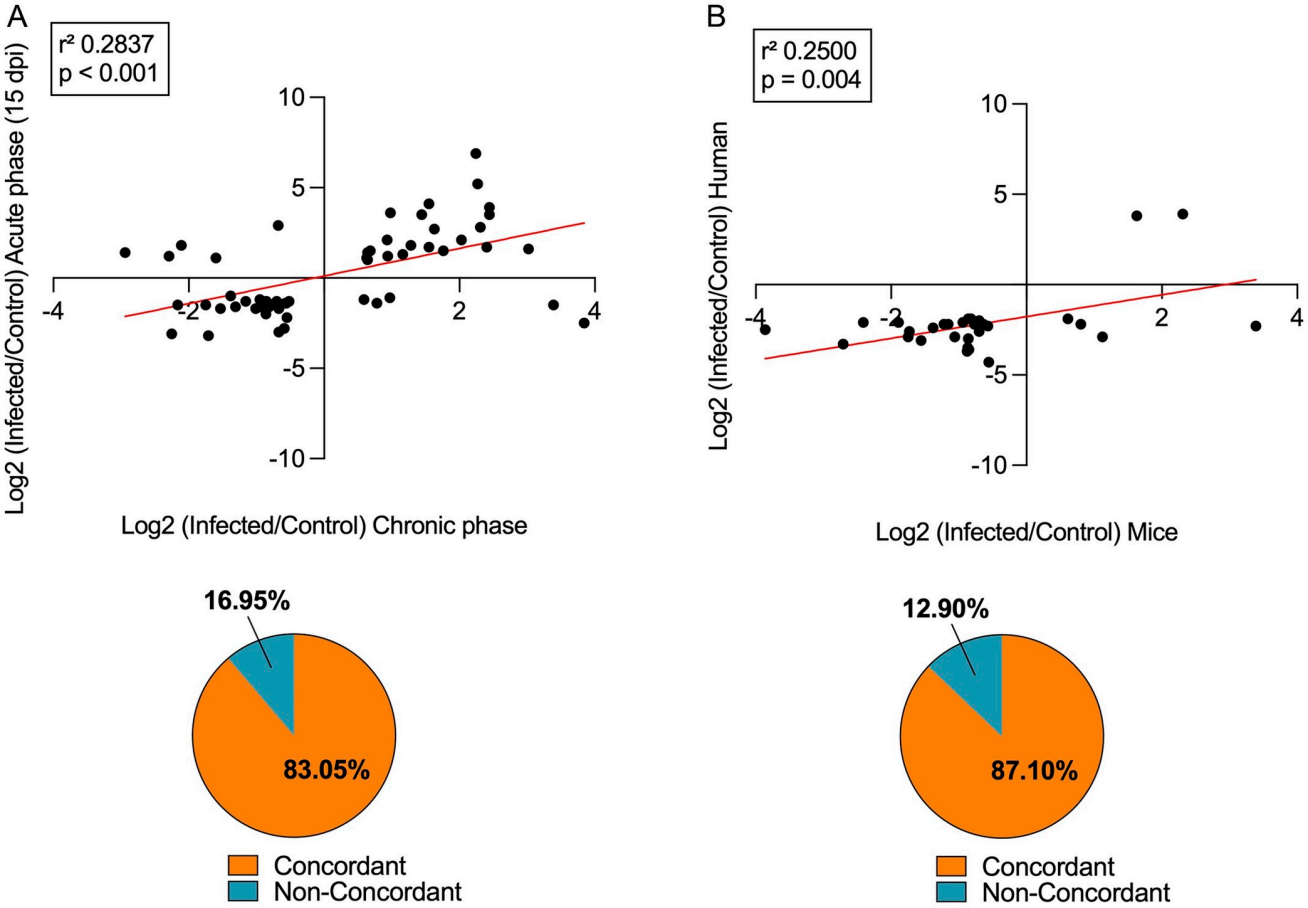
Concordance of miRNA transcriptome with previous studies. (A) Scatter plot of altered miRNAs (1.5-fold change) in mice cardiac tissue in the chronic phase (this study) and in mice cardiac tissue in the acute phase with a pie chart of direction agreements. (B) Scatter plot of altered miRNAs (1.5-fold change) in mice cardiac tissue in the chronic phase (this study) and in cardiac tissue of patients with end-stage CCC with a pie chart of direction agreements.

DEMs within the vehicle-treated group were identified by comparing those with the noninfected controls. DEMs with 1.5-fold change (up- or downregulated) from *T*. *cruzi*-infected group (Fig 3A), were subjected to functional analysis using Ingenuity Pathway Analysis Software (QIAGEN). Target filter analysis revealed putative targets for each RNA, while core analysis displayed the relevant regulatory pathways related to diseases and disorders. “Organismal injury and abnormalities” appeared with the highest number of miRNAs involved with best p-values, followed by “reproductive system disease”, “cancer”, “neurological disease”, and “psychological disorders” (Fig 3B). Molecular and cellular functions revealed “cellular development”, followed by “cellular growth and proliferation”, “cellular movement”, “cell cycle”, and “cell death and survival”, all these pathways are closely related to the cardiac remodeling process involved in CCC injury establishment (Fig 3C). Physiological system development and function revealed relevant pathways related to CCC such as “organ development”, “organismal development”, and “skeletal and muscular system development and function”, although with a smaller p-values, involved a higher number of miRNAs involved in CCC onset (Fig 3D). A more narrowed pathway analysis related to cardiotoxicity was also evaluated, revealing relevant pathways such as “cardiac dilatation”, “cardiac enlargement”, “cardiac fibrosis”, “cardiac inflammation”, and in a smaller scale, “cardiac infarction”, revealing that most DEMs are related to common cardiac impairments previously related to CCC (Fig 3E). Correlation analysis was done with previous studies to corroborate that the DEMs found in this study were modulated in other models of CD. A previous study analyzed the miRNA transcriptome profiling in the hearts of C57BL/6 mice with acute CD [38]. We observed a significant correlation of the DEMs in that study to our present findings (r^2^ = 0.2837, p < 0.001; Fig 4A), with an 83% agreement of the matching miRNAs in both studies altered in the same direction, suggesting that there are similar mechanisms involved in these two models. Another correlation analysis was done between our study and a miRNA transcriptome profiling in the cardiac tissue of patients with end-stage CCC [36]. We found a significant correlation between the two studies (r^2^ = 0.2500, p = 0.004; Fig 4B) with an 87% agreement of altered miRNAs in both studies, demonstrating that they regulated similarly and share, to some extent, underlying backgrounds to gene expression related to CCC progression and establishment in both mice and humans.

### Bz and Bz+PTX therapies are linked to the alteration of several miRNAs involved in CCC establishment pathways

**Fig 5:**
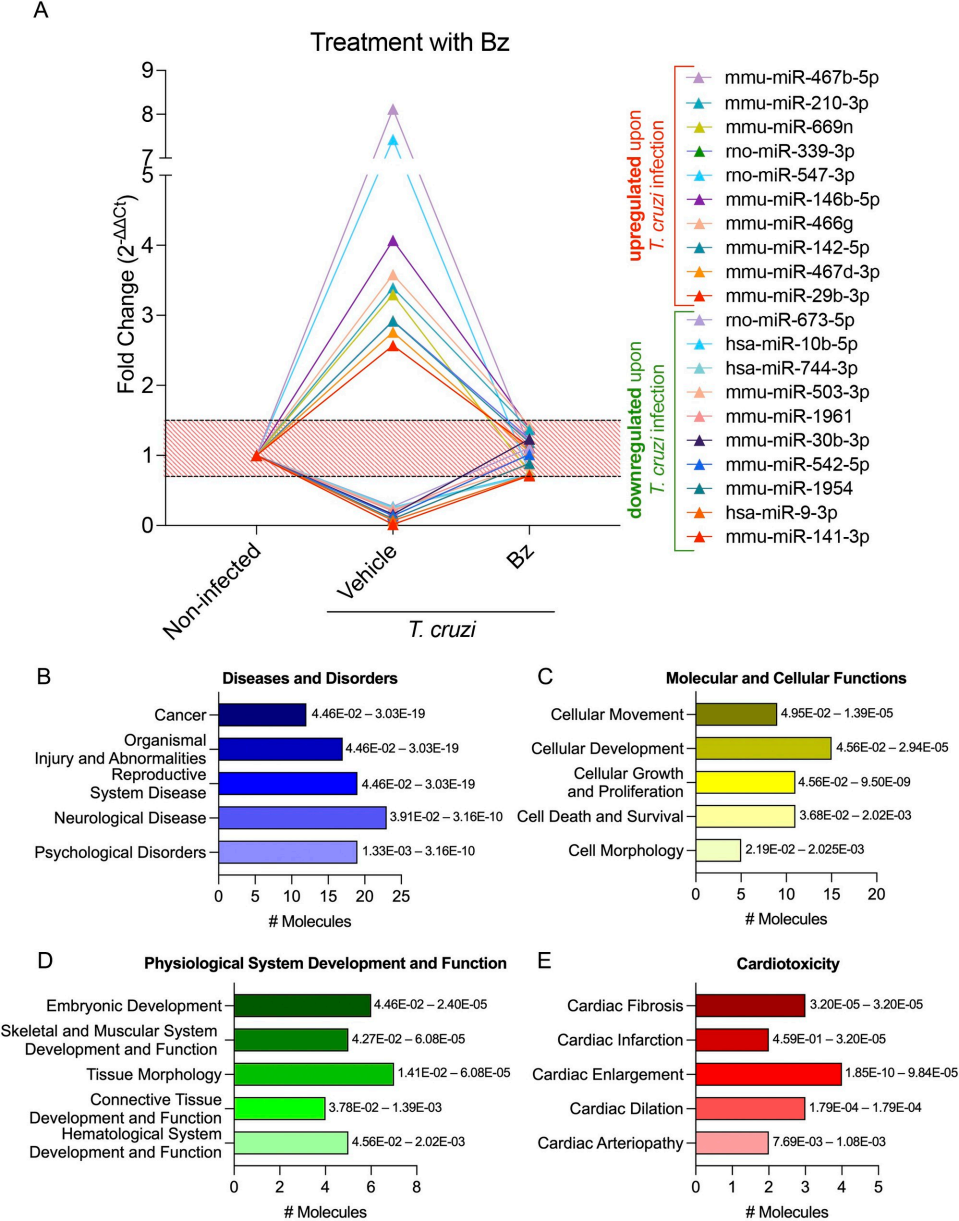
Characterization of immune response expression profile for the Bz group. (A) Expression of miRNAs altered in the infected group and with a restored expression upon Bz therapy. Relative expression was expressed as fold change (2^-ΔΔCt^). miRNAs with a fold change greater than 1.5 in the vehicle group and at the 0.5–1.5 range (red striped area) in the Bz therapy group were considered to have a restored expression. They were selected for further analysis on IPA software. (B-E) Activated pathways in the infected group with a restored expression upon Bz therapy. (B) Diseases and disorders pathway analysis. (C) Molecular and cellular function pathway analysis. (D) Physiological system development and function pathway analysis. (E) Cardiotoxicity pathway analysis. Significance intervals of enrichment are shown on the right of the bars.

**Fig 6:**
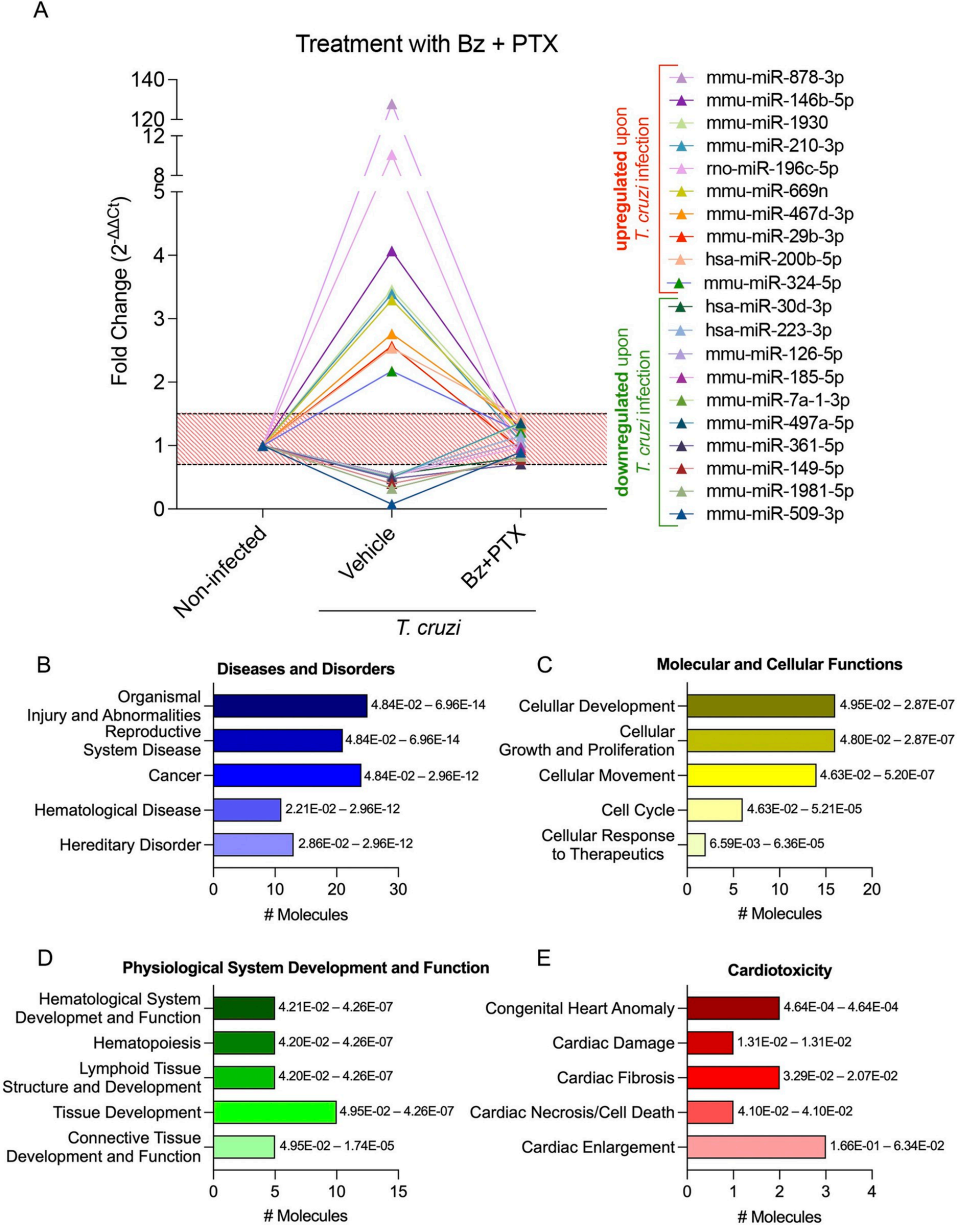
Characterization of immune response expression profile for the Bz+PTX group. (A) Expression of miRNAs altered in the infected group and with restored expression upon Bz+PTX therapy. Relative expression was expressed as fold change (2^-ΔΔCt^). miRNAs with a fold change greater than 1.5 in the vehicle group and at the 0.5–1.5 range (red striped area) in the Bz therapy group were considered to have a restored expression. They were selected for further analysis on IPA software. (B-E) Activated pathways in the infected group with a restored expression upon Bz therapy. (B) Diseases and disorders pathway analysis. (C) Molecular and cellular function pathway analysis. (D) Physiological system development and function pathway analysis. (E) Cardiotoxicity pathway analysis. Significance intervals of enrichment are shown on the right of the bars.

Following the miRNA transcriptome profiling characterization of the vehicle-treated group, we analyzed the miRNAs restored after Bz therapy (in between 1.5-fold change), aiming to dissect relevant signaling pathways affected by the treatment. As shown in S2 Table, 68 miRNAs were restored, among them the 10 most up- and 10 most downregulated miRNAs altered in *T*. cruzi infection (Fig 5A). The main affected diseases and disorders pathways were “cancer”, “organismal injury and abnormalities”, and “hematopoiesis” (Fig 5B). Related to molecular and cellular functions, the main affected pathways were “cellular development”, “cellular growth and proliferation” followed by “cellular movement”, “cell cycle”, and “cell death and survival” (Fig 5C). As to physiological system development and function, the modulated miRNAs affected mainly pathways related to “embryonic development”, “skeletal and muscular system development and function”, followed by “tissue morphology”, “connective tissue development and function”, and “hematological system development and function” (Fig 5D). Finally, the targeted analysis regarding cardiotoxicity revealed miRNA-affected pathways related to “cardiac fibrosis”, “cardiac infarction”, “cardiac enlargement”, “cardiac dilation”, and “cardiac arteriopathy” (Fig 5E). In the Bz+PTX group, 58 miRNAs were restored after therapy (S3 Table), among them the 10 most up- and 10 most downregulated ones altered upon *T*. cruzi infection (Fig 6A). The combined therapy showed distinct regulated pathways from the Bz therapy alone, as regarding diseases and disorders the main affected pathways were “organismal injury and abnormalities”, “reproductive system disease”, followed by “cancer”, “hematological disease”, and “hereditary disorder” (Fig 5B). The modulated miRNAs affected molecular and cellular functions mainly related to “cellular development”, “cellular growth and proliferation”, followed by “cellular movement”, “cell cycle”, and “cellular response to therapeutics” (Fig 5C). As to the main affected physiological system development and function pathways, we found “hematological system development and function”, “hematopoiesis”, “lymphoid tissue structure and development”, “tissue development”, and “connective tissue development and function” as the most regulated pathways upon Bz+PTX therapy (Fig 5D). Finally, related to cardiotoxicity, Bz+PTX therapy affected pathways were mainly related to “congenital heart anomaly”, “cardiac damage”, “cardiac fibrosis”, “cardiac necrosis/cell death”, and “cardiac enlargement” (Fig 5E).

### Target prediction analysis revealed a relevant participation of miR-146b-5p in CCC inflammation establishment, which was restored by Bz or Bz+PTX therapy

**Fig 7:**
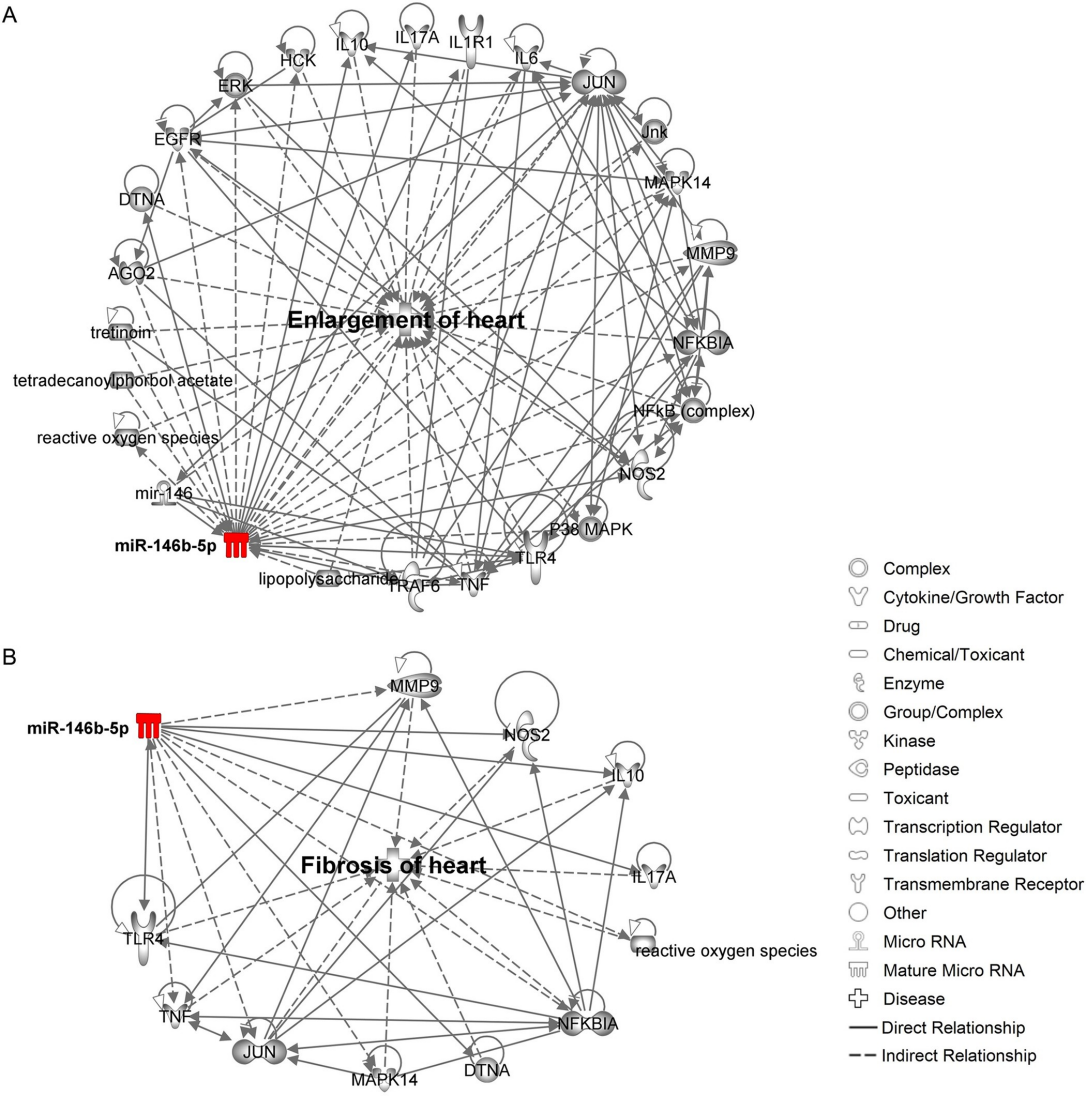
Target prediction analysis for Bz-treated group. (A) Enlargement of heart pathway. (B) Fibrosis of heart pathway. The red color indicates miRNA upregulation. Full lines indicate a direct relationship, while dashed lines indicate an indirect relationship.

Aiming to dissect the miRNA-mRNA interactions contributing to some key pathways affected by therapy, we selected some cardiotoxicity pathways to look into more closely. From the Bz group, we elected two affected networks related to “enlargement of heart” (Fig 7A) and “fibrosis of heart” (Fig 7B), which are one of the main pathological processes involved in the onset of CCC found in the cardiotoxicity analysis. From all the Bz-restored miRNAs used as input for this analysis, miR-146b-5p emerges in both processes as a key miRNA, promoting direct regulation of relevant genes such as *DTNA* (dystrobrevin-alpha), *IL-10* (interleukin-10), *IL-17A* (interleukin-17A), *NOS2/iNOS* (nitric oxide synthase 2/inducible nitric oxide synthase), *TLR4* (toll-like receptor 4) and *TRAF6* (TNF-receptor associated factor 6). Additionally, this miRNA also affects indirectly some relevant genes such as *EGFR* (epidermal growth factor receptor), *ERK* (serine/threonine protein kinase), *IL-6* (interleukin-6), *Jnk* (c-JUN N-terminal kinase), *MMP9* (matrix metallopeptidase 9), *NFKBIA* (nuclear factor kappa-B inhibitor alpha) and *TNF* (tumor necrosis factor), contributing to the microenvironment establishment of the cardiac tissue remodeling leading to cardiomegaly traditionally seen in CCC.

**Fig 8:**
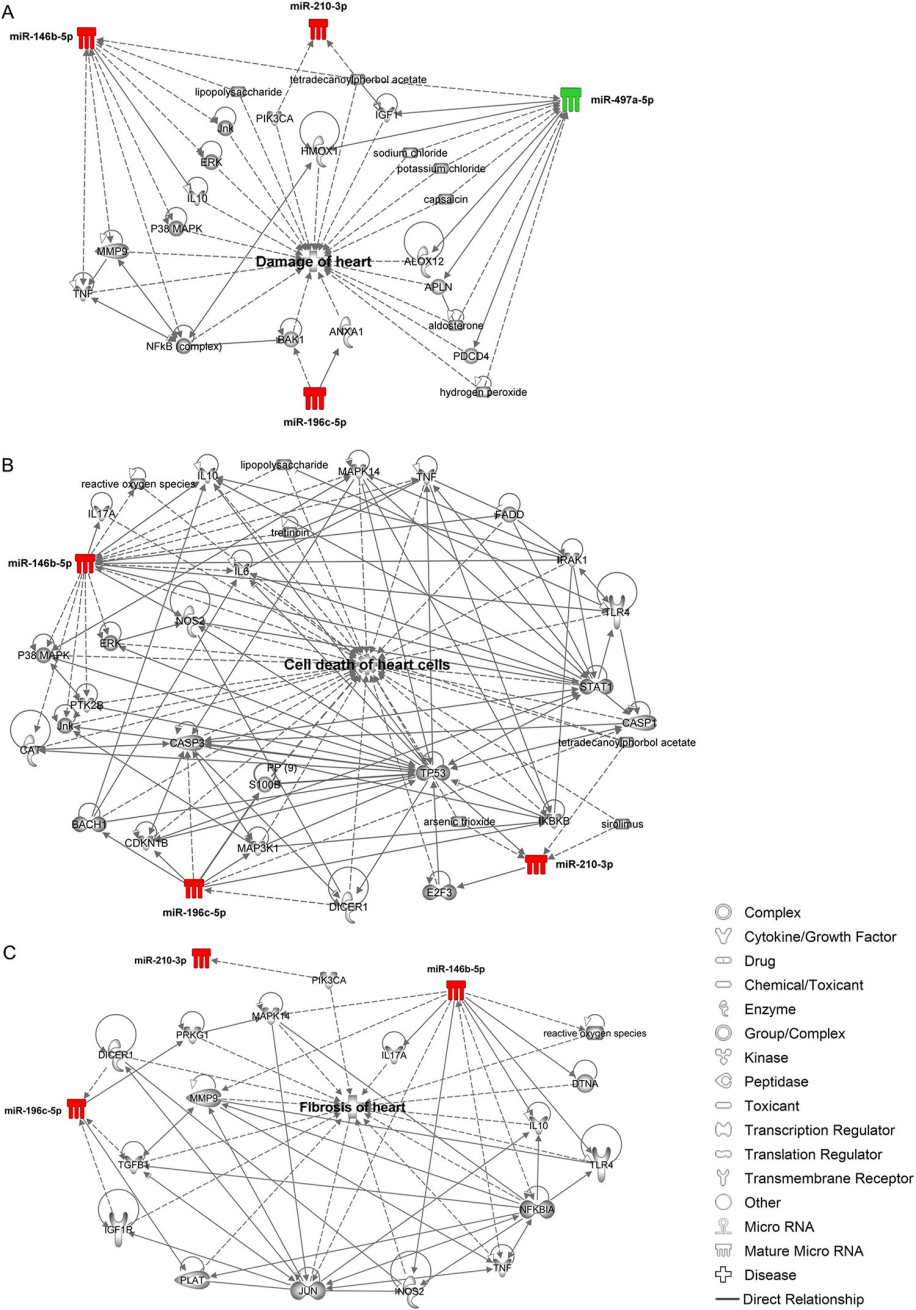
Target prediction analysis for Bz+PTX treated group. (A) Damage of heart pathway. (B) Cell death of heart cells pathway. (B) Fibrosis of heart pathway. The red color indicates miRNA upregulation, while the green color indicates miRNA downregulation. Full lines indicate a direct relationship, while dashed lines indicate an indirect relationship.

**Fig 9:**
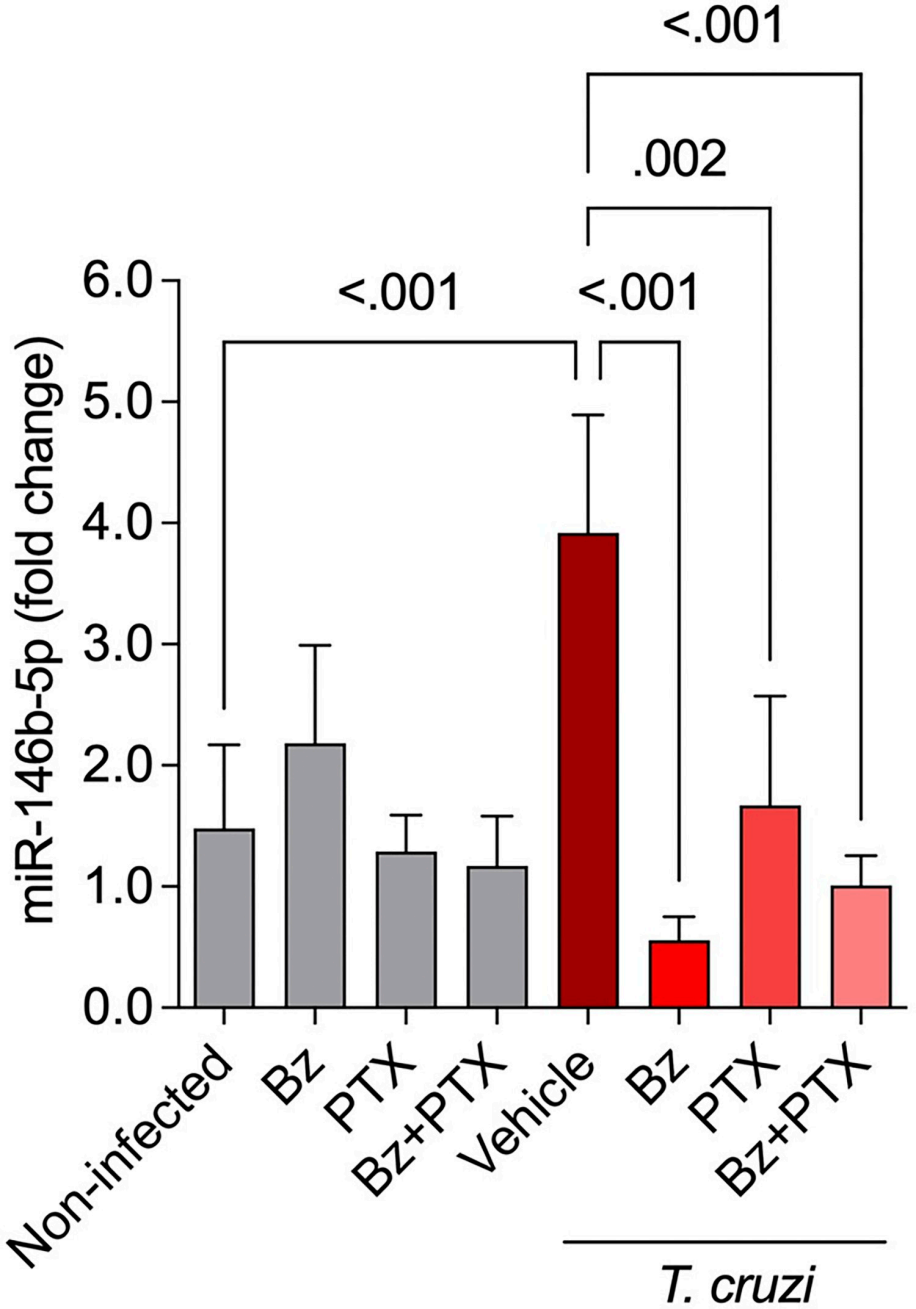
Validation of miR-146b-5p expression in individual samples. miR-146b-5p was assessed by real-time RT-qPCR in each experiment group, with groups of 3–5 animals per group. The expression is shown as mean ± SD for each group by 2^-ΔΔCt^ relative quantification method. Groups were compared using unpaired one-way ANOVA with Tukey’s multiple comparisons with a 95% confidence level.

Conversely, the combined Bz+PTX therapy revealed other key restored miRNAs like miR-196c-5p, miR-210-3p, and miR-497a-5p that were involved in the regulation of genes related to “damage of heart” (Fig 8A), “cell death of heart cells” (Fig 8B) and “fibrosis of heart” (Fig 8C) pathways. In the “damage of heart” pathway, miR-146b-5p was found to be directly targeting *IL-10* and indirectly affecting *TNF*, *MMP9*, *ERK* and *Jnk*. miR-196c-5p directly targeted *ANXA1* (annexin A1) and indirectly targeted *BAK1* (BCL2 antagonist/killer 1). MiR-210-3p appeared in the network as controlled by other genes such as *PIK3CA* (phosphatidylinositol-4,5-biphosphate-3-kinase catalytic subunit alpha) and *IGF1* (insulin-like growth factor 1) and, finally, miR-497a-5p, which was downregulated under *T*. *cruzi* infection, appeared to directly target other genes that may be involved in CCC establishment, such as *ALOX12* (arachidonate 12-lipoxygenase), *APLN* (apelin) and *PDCD4* (programmed cell death 4). Additionally, in the “cell death of heart cells” network, miR-146b-5p revealed direct targeting of cell death-related molecules such as *FADD* (Fas associated via death domain) and indirect targeting of *PTK2B* (protein tyrosine kinase 2-beta) and CAT (catalase). miR-196c-5p revealed direct targeting of *CDKN1B* (cyclin dependent kinase inhibitor 1B), *IKBKB* (inhibitor of nuclear factor kappa B kinase subunit alpha) and indirect targeting of relevant cell death molecules as *CASP1* (caspase 1) and *CASP3* (caspase 3). miR-210-3p also appeared in this network and was found to be directly targeting *E2F3* (E2F transcription factor 3). Finally, in the “fibrosis of heart” network, miR-146b-5p appeared once again, directly targeting *DTNA*, *IL-10*, *TLR4* and *IL-17A*, and miR-196c-5p revealed *PRKG1* (protein kinase CGMP-dependent 1) and *PLAT* (plasminogen activator, tissue type). These miRNA-mRNAs networks demonstrate how complex and multifactorial the pathogenesis of CCC is, revealing miR-146b-5p in the core of all cardiotoxicity networks analyzed in this study. Therefore, miR-146b-5p was selected for further validation of our microarray results, based on previous studies done in the same C57BL/6 model in the acute phase [38] that were also seen in this study as upregulated and affected by Bz and Bz+PTX therapies. miR-146b-5p showed upregulation in expression levels in the vehicle treated group (3.92 ± 0.97; p < 0.001) compared with the noninfected controls (1.48 ± 0.69), and interestingly Bz (0.56 ± 0.19; p < 0.001), PTX (1.67 ± 0.90; p = 0.002) and Bz+PTX (1.01 ± 0.24; p < 0.001) therapies were able to reverse the upregulation of this miRNA compared with the vehicle treated group (Fig 9).

## Discussion

Efforts have been made to understand miRNA molecular pattern and affected pathways behind CCC establishment and progression [35,36,38,48]; however, the molecular mechanisms underpinning CCC remain unsolved and to date there is no study focusing on the regulation of miRNA expression upon etiological treatment with Bz and, especially, with an associated immunomodulatory treatment. Here, we show the miRNA profiling in the hearts of C57BL/6 mice chronically infected with *T*. *cruzi* and, for the first time, we reveal how the etiological treatment using a suboptimal dose of Bz, alone or in association with the immunomodulatory agent PTX, regulate the miRNA network in the heart affected by *T*. *cruzi* infection.

The experimental infection with *T*. *cruzi* Colombian strain in C57BL/6 mice used here is a well-established model used in several previous studies [25,26,40–42,49,50], reproducing key ECG alterations found in human CCC, such as bradycardia, arrhythmias, prolonged P wave, PR and QTc intervals, AVB2, and other major traits of CCC (e.g., fibrosis) [9,51], Therefore, with an adequate model to reproduce main features of CCC, we focused on the effects of Bz, PTX, and the combined Bz+PTX therapy in this model. Our data showed that Bz, PTX, and Bz+PTX therapies reduced the percentage of mice afflicted with ECG abnormalities, arrhythmias, AVB2, and AVB2 events in two-minute records as seen in previous studies [25,26]. Moreover, suboptimal dose of Bz was able to reduce parasite load in the heart tissue as previous seen in study done *in vitro* and *in vivo* where lower doses of Bz were able to control parasite burden as efficiently as the usual full dose (100 mg/Kg/day) [16]. Therapy with PTX alone showed no effect on parasite load in the hearts of *T*. *cruzi*-infected mice, which was expected as PTX has no direct trypanocidal activity described in the literature, although it indirectly disfavored TNF-induced astrocyte infection by *T*. *cruzi*, reducing TNFR1 expression [52] and was able to reverse critical ECG abnormalities, corroborating previous studies [25,26]. Finally, therapy with combined Bz+PTX was also able to reverse relevant ECG changes and most importantly, did not hamper Bz trypanocidal activity [26]. Additionally, a previous study using the same experimental model and treatment, followed the animals for 30–50 days after treatment discontinuation and showed that most ECG abnormalities were completely or partially re-established in the groups treated with Bz or PTX alone, whereas Bz+PTX treatment completely sustained the reversion of these cardiac abnormalities [26]. Taken together, our findings reported a suitable CCC murine model reflecting the same cardiac alterations in humans, and most importantly the Bz, PTX and Bz+PTX therapies tested here were able to restore relevant heart electrophysiological parameters.

MicroRNA transcriptome profiling was previously done in the heart of mice with acute *T*. *cruzi* infection [38,48], in murine thymic epithelial cells [37], and in the heart of patients with end-stage CCC subjected to heart transplant [36]. Our miRNA transcriptome profiling in murine hearts with CCC revealed a vast change in the molecular profile, showing several miRNAs involved in immune dysregulation and CCC progression, as previously observed in other studies [36,38,48,53]. When compared with a previous study done in the acute phase (15–45 dpi) [38], our current study showed an increase in the amount of differentially expressed miRNAs as the disease progresses to its chronic stage. The acute phase study was done in the same experimental model (C57BL/6 mice infected with *T*. *cruzi* Colombiana strain) showed 19 altered miRNAs at 15 dpi, 66 at 30 dpi, and 96 at 45 dpi [38]. In contrast, we found 221 altered miRNAs at 150 dpi, suggesting that CD progression is causing wide molecular alterations in the cardiac tissue, directly promoting dysregulation of miRNAs. On the one hand, our data showed miR-133a (0.4-fold change) and miR-133b (0.7-fold change) downregulation in the chronic mouse model, corroborating previous studies analyzing a set of miRNAs in cardiac tissue from patients with end-stage CCC subjected to heart transplant [35]. On the other hand, we observed miR-203 (5.4-fold change), miR-146a (5.0-fold change), miR-146b (4.1-fold change), miR-155 (3.1-fold change), miR-20b (4.3-fold change), miR-21 (1.6-fold change), miR-142-5p (2.9-fold change), miR-142-3p (5.4-fold change), and miR-182 (15.8-fold change) upregulation. Conversely, miR-322 (0.2-fold change), miR-149 (0.4-fold change), miR-503 (0.2-fold change), miR-139-5p (0.6-fold change), and miR-145 (0.6-fold change) showed downregulation. These findings agree with previous study using the same mouse model in the acute phase (at 15, 30, and 45 dpi), which selected differentially expressed miRNAs for further analysis based on their correlation with parasitemia levels and QTc interval prolongation [38]. Additionally, we found an 83% agreement between the altered miRNAs in the acute and chronic phase (at 150 dpi), meaning they vary in the same direction, implying that the miRNA expression differences are precociously established in *T*. *cruzi* acute infection, and suggesting a slight change in miRNA expression pattern as the disease progresses.

Another correlation analysis was done with our data on miRNA in the CCC model and the miRNA transcriptome profiling in the heart of patients with end-stage CCC [36]. In this case, although only a small percentage of differentially expressed miRNAs were the same, they showed even higher concordance, suggesting that CCC onset in murine model and humans share common molecular backgrounds. Recently, the evaluation of 88 miRNAs in the heart of chronically *T*. *cruzi*-infected mice and serum samples of CCC patients found three overlapping upregulated miRNAs, miR-21-5p, miR-29b-3p, and miR-29b-3p [54]. Out of the three, two miRNAs, miR-21-5p (1.6-fold change) and miR-29b-3p (2.6-fold change) also showed upregulation in our study, supporting that our model of CCC reproduces relevant aspects of Chagas heart disease and agrees with previous miRNA profiling studies done in a wide range of biological samples and using different experimental models [36,38,48,53].

Next, the effect of Bz and Bz+PTX therapy on the regulation of altered miRNAs was evaluated. Bz therapy showed regulation of promising up- and downregulated miRNAs that play a pivotal role in CCC onset/progression. For instance, miR-467b has been implicated in the regulation of atherosclerosis and secretion of the proinflammatory cytokines IL-6, IL-1β, and TNF [55,56], which are also knowingly dysregulated in CCC [57]. Conversely, miR-142-5p overexpression seem to contribute to the establishment of chronic inflammatory diseases, sustaining profibrogenic properties of macrophages induced by IL-4 and IL-13 and showing upregulation in macrophages from tissue samples of patients with idiopathic pulmonary fibrosis [58]. Among downregulated miRNAs affected by Bz therapy, we found miR-141-3p, which is involved in the regulation of mitochondrial dysfunction [59] and oxidative stress, since inhibition of this miRNA in hypoxia-induced cardiomyoblasts increased cell viability and reduced apoptosis mainly by affecting the PI3K/AKT pathway [60]. Target prediction analysis for the Bz therapy group revealed miR-146b-5p in the core of two main processes related to heart enlargement and fibrosis. We demonstrated that Bz therapy was able to reverse the upregulation of this miRNA, possibly reverting the downregulation of its predicted targets. Among the genes targeted by miR-146b-5p, we can highlight the participation of several genes related to cell growth and proliferation such as *EGFR*, *ERK*, and *Jnk*, and MAPK signaling pathway, previously shown to be related to *T*. *cruzi* evasion from the immune system [61], tissue remodeling such as *DTDNA*, related to muscular dystrophies, and *MMP9*, involved in the breakdown of extracellular matrix, degrading type IV and V collagen, with relevant participation in CCC onset [62,63]. Additionally, relevant immune system genes were also revealed in the target prediction analysis such as *IL-10*, *NFKB1A*, *NOS2/iNOS*, *TLR4*, *TNF*, and *TRAF6*, previously shown to be related to *T*. *cruzi*-triggered inflammatory process, production of cytokines and CCC onset [64]. Nevertheless, in a recent study, we demonstrated upregulation of IL-10 upon Bz therapy [64], which now based on our current miRNA data we suggest might be through miR-146b-5p upregulation, although further confirmation is needed.

Although Bz has been used as a trypanocidal agent for over 50 years [14,65], its immunomodulatory properties have not been unveiled until recently [16,66–69]. First, it has been shown that Bz treatment significantly reduced production of nitrite, IL-6, IL-10 and, partially, TNF and IL-1β in LPS-stimulated murine macrophages [69], which was further confirmed in *T*. *cruzi*-infected mice [68]. Second, the potential role of Bz in reducing peripheral blood leukocytes and gene expression of TNF and NOS2/iNOS via NF-κB and MAPK inhibition has been described in a model of cecal sepsis [66]. Furthermore, attenuation of the inflammatory response due to nuclear factor erythroid 2–related factor 2 (NRF2) activation in the liver and an increase in antioxidant defenses, with TLR4-signaling inhibition, were detected in the same model [67]. We recently showed a vast alteration of mRNA expression caused by *T*. *cruzi* infection that is restored upon treatment with a suboptimal dose of Bz in the CCC model used in our current study, supporting a regulation of the immune response after etiological treatment [64]. All these data led us to propose that the attenuation of inflammation caused by Bz, due to its intrinsic immunomodulatory properties or simply by killing the parasite, may mitigate the subsequent cardiac damage caused by the long-term inflammation process, which may represent the main factor involved in the modulation of certain miRNAs and pathways in experimental CCC.

Bz+PTX combined therapy showed a few distinctly altered miRNAs, including miR-669n, which is related to increased production of TNF, IL-6, and IFN-γ by targeting *SENP6* protein in mouse macrophages [70]. Among the downregulated miRNAs, Bz+PTX reduced miR-509-3p, a miRNA previously seen to be involved in cell proliferation and migration processes in renal carcinoma cells [71]. Other relevant regulated miRNA is miR-149-5p, whose downregulation stimulates proliferation, invasion, and migration of vascular smooth muscle cells, acting as prognostic factor for survival of human sarcoma [72]. Additionally, miR-497a-5p was found downregulated in LPS-induced inflammatory reactions, targeting the IRAK2/NF-κB pathway [73]. Our predictive study supports a role for miR-146b-5p targeting genes related to immune response, cell proliferation/apoptosis, through the MAPK kinase pathway as *ERK*, *Jnk*, *PTK2B*, and *FADD*, involved in cell apoptotic mechanisms, and *CAT*, related to antioxidant response relevant for *T*. *cruzi* infection establishment [74]. Conversely, other miRNAs, as miR-196c-5p, has known anti-inflammatory properties, targeting relevant cell cycle and death processes through downregulation of *BAK1*, *CDNKB1*, *CASP3*, *CASP1*, *IκBκB*, and *ANXA1*. These results show that the combined Bz+PTX therapy acts on a multitude of miRNAs involved in crucial pathophysiological features related to CCC. Beyond its hemorheological phosphodiesterase inhibitor activity, PTX also exerts immunomodulatory properties [22,75]. It has been shown in a CCC model that PTX reduced the number of perforin-positive cells invading the cardiac tissue and delayed the progression of heart injury, reversing ECG abnormalities and restoring left ventricle ejection fraction [25]. The effects of Bz+PTX therapy on CCC were also previously explored by our group, supporting reduction in myocarditis and fibrosis, improvement of ECG abnormalities, reduction of TNF, and TNFR1 expression on CD8^+^ T cells and, more importantly, these effects were sustained after 30 days of treatment suspension [26]. Additionally, we recently assessed miR-146b-5p expression in *T*. *cruzi*-infected H9C2 cardiac cells, showing upregulation of this miRNA in early hours of infection with expression restoration upon Bz+PTX therapy, corroborating the participation of this miRNA in the parasite-host interaction process [76].

Lastly, we validated miR-146b-5p in individual samples, based on differential expression found in the acute phase of *T*. *cruzi* infection [38], crucially shown in *in vitro* model of *T*.*cruzi*-cardiomyocyte interaction [76]. miR-146b-5p was found upregulated in acutely infected mice [38] and here in the vehicle-treated infected group, supporting the consistent effect of infection on miR-146b-5p expression in both acute and chronic phases of CD. In this case, Bz and the combined Bz+PTX therapy restored the miRNA expression to levels found in noninfected age-matched controls, suggesting that the therapies used here somehow influence the expression of this miRNA. The miR-146b-5p expression highly depends on inflammatory stimulus, especially proinflammatory cytokines such as IL-1β, TNF, and IFN-γ [77]. Furthermore, *NF-κB*, *C/EBPβ*, *c-Fos*, and *STAT3/6* were identified as targets of miR-146b-5p [78]. Thus, these data suggest that the production of proinflammatory cytokines, intrinsic to CD pathogenesis [57,79], might influence the upregulation of miR-146b-5p, as seen in our recent study [64]. Moreover, the downmodulation of miR-146b-5p upon Bz and Bz+PTX therapies might be due to the parasite clearance and/or the immunomodulatory effects previously reported individually for both Bz [66,68,69,80] and PTX [75,81,82]. Thus, this result opens a new venue for the further analysis of miR-146b-5p as a potential biomarker of cure in CD since the restored level of this miRNA occurred in parallel with parasite clearance after treatment and downregulation of inflammatory mediators, such as TNF/TNFR1 and NO/iNOS pathways, involved in CCC pathogenesis, as previously shown [25,26].

This study assessed the miRNA profiling in CCD under an etiological and immunomodulatory therapy, opening opportunities for molecular approaches and therapeutic interventions in CD. We recognize that our study was not able to contemplate all the experiments needed for a full understanding of the altered miRNAs value in the context of CCC progression and establishment. For instance, serum miRNAs were not assessed to correlate with the alterations in the cardiac tissue, which could be useful for identifying urgently needed biomarkers for early assessment of therapeutic outcomes in CD [83,84]. However, the altered miRNAs restored after a suboptimal dose of Bz and the combined Bz+PTX therapies suggest a pronounced relationship between etiological and immunomodulatory treatment in various molecular pathways. Finally, the altered miRNAs identified in this study could possibly serve as drug targets or auxiliary therapy, although more research is necessary to evaluate the effects of specific miRNAs in CD progression.

## Supporting information

S1 Table Up- or downregulated microRNAs (1.5-fold change) in the vehicle-treated group. (DOCX)

S2 Table microRNAs restored to levels of uninfected mice (between 1.5-fold-change) after Bz treatment. (DOCX)

S3 Table microRNAs restored to levels of uninfected mice (between 1.5-fold-change) after Bz+PTX treatment. (DOCX)
