# Supplementary material for: Treatment with benznidazole and pentoxifylline regulates microRNA transcriptomic profile in a murine model of Chagas chronic cardiomyopathy
Source: PLoS Negl Trop Dis. 2023 Mar 27;17(3):e0011223. doi: 10.1371/journal.pntd.0011223 (PMC10121046; doi:10.1371/journal.pntd.0011223)
Supplement: S1 Table — (DOCX) [file pntd.0011223.s001.docx]

**Supplementary table 1.** Up- or downregulated microRNAs (1.5-fold change) in the vehicle-treated group.

| **Name** | **Acession Number** | **Infected** | **Bz** | **Bz+PTX** |
| --- | --- | --- | --- | --- |
| mmu-miR-1959 | MIMAT0009432 | 1139.488 | 514.541 | 418.923 |
| mmu-miR-470-3p | MIMAT0004760 | 585.3900146 | 3.608999968 | 0.041000001 |
| mmu-miR-878-3p | MIMAT0004933 | 127.887001 | 3.227999926 | 1.351999998 |
| mmu-miR-1982-3p | MIMAT0009460 | 58.125 | 65.11599731 | 5.756000042 |
| mmu-miR-297c-5p | MIMAT0004865 | 31.88199997 | 9.324000359 | 926.0930176 |
| mmu-miR-196a-2-3p | MIMAT0004618 | 26.70899963 | 104.6179962 | 31.01099968 |
| mmu-miR-467e-5p | MIMAT0005293 | 20.67900085 | 5.223999977 | 42.61100006 |
| mmu-miR-326-3p | MIMAT0000559 | 15.97799969 | 3.536999941 | 11.99699974 |
| mmu-miR-199b-5p | MIMAT0000672 | 15.93500042 | 18.34000015 | 21.48699951 |
| mmu-miR-182-5p | MIMAT0000211 | 15.85299969 | 6.308000088 | 4.875999928 |
| rno-miR-190b-5p | MIMAT0005302 | 14.35700035 | 4.46999979 | 5.138999939 |
| mmu-miR-883a-3p | MIMAT0004849 | 13.69299984 | 0.444999993 | 0.225999996 |
| mmu-miR-337-5p | MIMAT0004644 | 12.15100002 | 29.92300034 | 102.9440002 |
| rno-miR-20b-5p | MIMAT0003211 | 10.49499989 | 25.96999931 | 16.4279995 |
| mmu-miR-698-3p | MIMAT0003488 | 10.13899994 | 5.284999847 | 4.524000168 |
| rno-miR-196c-5p | MIMAT0005303 | 10.1260004 | 0.135000005 | 0.773999989 |
| mmu-miR-599 | MIMAT0012772 | 8.737000465 | 0.233999997 | 5.495999813 |
| hsa-miR-197-3p | MIMAT0000227 | 8.204000473 | 22.63199997 | 37.91699982 |
| mmu-miR-467b-5p | MIMAT0005448 | 8.119999886 | 1.129999995 | 5.899000168 |
| rno-miR-547-3p | MIMAT0012851 | 7.427000046 | 0.758000016 | 1.972000003 |
| mmu-miR-711 | MIMAT0003501 | 7.163000107 | 4.867000103 | 0.159999996 |
| rno-miR-24-1-5p | MIMAT0003153 | 6.821000099 | 19.38699913 | 15.06200027 |
| mmu-miR-141-5p | MIMAT0004533 | 6.627999783 | 18.41699982 | 0.071000002 |
| mmu-miR-1897-5p | MIMAT0007864 | 6.440999985 | 19.26099968 | 9.095000267 |
| mmu-miR-101a-5p | MIMAT0004526 | 5.874000072 | 4.355999947 | 2.121000051 |
| mmu-miR-1192 | MIMAT0005850 | 5.668000221 | 3.174999952 | 2.792999983 |
| mmu-miR-203-3p | MIMAT0000236 | 5.44299984 | 2.894999981 | 3.526000023 |
| mmu-miR-142-3p | MIMAT0000155 | 5.43900013 | 2.154000044 | 2.073999882 |
| mmu-miR-467a-5p | MIMAT0003409 | 5.284999847 | 2.194999933 | 4.236999989 |
| mmu-miR-146a-5p | MIMAT0000158 | 4.960999966 | 2.062000036 | 1.567999959 |
| mmu-miR-18a-5p | MIMAT0000528 | 4.829999924 | 2.737999916 | 2.717999935 |
| mmu-miR-20b-5p | MIMAT0003187 | 4.737999916 | 2.483999968 | 4.002999783 |
| mmu-miR-511-5p | MIMAT0004940 | 4.619999886 | 0.469999999 | 3.532000065 |
| mmu-miR-146b-5p | MIMAT0003475 | 4.070000172 | 1.396000028 | 1.222000003 |
| mmu-miR-146b-5p | MIMAT0005845 | 3.992000103 | 2.602999926 | 1.886000037 |
| mmu-miR-362-5p | MIMAT0000706 | 3.661000013 | 5.943999767 | 5.369999886 |
| mmu-miR-466g | MIMAT0004883 | 3.582000017 | 1.424999952 | 1.521000028 |
| mmu-miR-340-3p | MIMAT0000586 | 3.467000008 | 2.621000051 | 3.023000002 |
| mmu-miR-1930 | MIMAT0009393 | 3.460000038 | 1.501000047 | 1.207000017 |
| mmu-miR-210-3p | MIMAT0000658 | 3.394000053 | 1.368999958 | 1.06099999 |
| mmu-miR-669n | MIMAT0009427 | 3.295000076 | 0.757000029 | 1.205000043 |
| mmu-miR-465b-5p | MIMAT0004871 | 3.23300004 | 2.842999935 | 3.448999882 |
| mmu-miR-342-5p | MIMAT0004653 | 3.13499999 | 0.314999998 | 0.167999998 |
| mmu-miR-155-5p | MIMAT0000165 | 3.088000059 | 0.582000017 | 1.779000044 |
| rno-miR-339-3p | MIMAT0004648 | 2.921000004 | 1.233999968 | 7.90199995 |
| mmu-miR-142-5p | MIMAT0000154 | 2.91899991 | 1.187000036 | 0.30399999 |
| mmu-miR-465a-5p | MIMAT0002106 | 2.905999899 | 2.382999897 | 1.953999996 |
| mmu-miR-467d-3p | MIMAT0004887 | 2.760999918 | 1.050999999 | 1.309999943 |
| mmu-miR-696 | MIMAT0003483 | 2.714999914 | 2.976999998 | 1.644000053 |
| mmu-miR-465c-5p | MIMAT0004873 | 2.687000036 | 0.338 | 0.140000001 |
| mmu-miR-598-3p | MIMAT0004942 | 2.657000065 | 2.263999939 | 0.573000014 |
| mmu-miR-1306-3p | MIMAT0009411 | 2.625999928 | 3.39199996 | 0.460000008 |
| mmu-miR-29b-3p | MIMAT0000127 | 2.569000006 | 1.120000005 | 0.939999998 |
| hsa-miR-200b-5p | MIMAT0004571 | 2.536999941 | 1.011000037 | 1.440999985 |
| mmu-miR-425-5p | MIMAT0004750 | 2.532000065 | 2.063999891 | 2.696000099 |
| mmu-miR-342-3p | MIMAT0000590 | 2.430000067 | 1.013000011 | 1.980999947 |
| mmu-miR-215-5p | MIMAT0000904 | 2.355000019 | 0.163000003 | 1.31099999 |
| mmu-miR-2183 | MIMAT0011287 | 2.323999882 | 1.324000001 | 3.499000072 |
| hsa-miR-324-3p | MIMAT0000762 | 2.322999954 | 3.926000118 | 2.651000023 |
| mmu-miR-1962 | MIMAT0009435 | 2.28399992 | 6.323999882 | 6.111000061 |
| mmu-miR-694 | MIMAT0003474 | 2.227999926 | 1.110999942 | 0.563000023 |
| mmu-miR-324-5p | MIMAT0000555 | 2.174000025 | 1.353000045 | 1.210999966 |
| mmu-miR-490-3p | MIMAT0003780 | 2.072999954 | 2.094000101 | 1.457000017 |
| mmu-miR-138-1-3p | MIMAT0004668 | 2.055999994 | 1.58099997 | 0.300999999 |
| mmu-miR-547-3p | MIMAT0003173 | 2.049000025 | 0.734000027 | 1.149000049 |
| rno-miR-351-5p | MIMAT0000608 | 2.025000095 | 0.463999987 | 1.45599997 |
| mmu-miR-2138 | MIMAT0011214 | 1.978000045 | 1.687999964 | 0.541999996 |
| rno-miR-350 | MIMAT0000604 | 1.976999998 | 4.551000118 | 1.11500001 |
| mmu-let-7d-3p | MIMAT0000384 | 1.968000054 | 6.502999783 | 3.924000025 |
| rno-miR-146b-5p | MIMAT0005595 | 1.967000008 | 1.279000044 | 1.126999974 |
| mmu-miR-331-3p | MIMAT0000571 | 1.958999991 | 0.393000007 | 1.906999946 |
| mmu-miR-1191a | MIMAT0005849 | 1.944000006 | 0.569999993 | 2.423000097 |
| mmu-miR-34b-3p | MIMAT0004581 | 1.919999957 | 0.68900001 | 0.716000021 |
| mmu-miR-133a-5p | MIMAT0003473 | 1.91900003 | 158.5529938 | 4.946000099 |
| mmu-miR-130b-3p | MIMAT0000387 | 1.909999967 | 2.138000011 | 2.27699995 |
| mmu-miR-1931 | MIMAT0009394 | 1.894000053 | 0.500999987 | 4.395999908 |
| rno-miR-29b-1-5p | MIMAT0005445 | 1.871000051 | 0.147 | 0.442000002 |
| mmu-miR-132-3p | MIMAT0000144 | 1.860000014 | 1.213000059 | 0.948000014 |
| mmu-miR-667-3p | MIMAT0003734 | 1.82099998 | 1.531999946 | 1.692999959 |
| mmu-miR-1188-5p | MIMAT0005843 | 1.792000055 | 1.348999977 | 2.503999949 |
| mmu-miR-223-3p | MIMAT0000665 | 1.784999967 | 1.167000055 | 1.583999991 |
| mmu-miR-680 | MIMAT0003457 | 1.743000031 | 0.323000014 | 2.282999992 |
| mmu-miR-296-5p | MIMAT0000374 | 1.741999984 | 1.43599999 | 1.465999961 |
| mmu-miR-339-3p | MIMAT0004649 | 1.72300005 | 1.144999981 | 2.039000034 |
| mmu-miR-139-3p | MIMAT0004662 | 1.700000048 | 0.600000024 | 2.25 |
| mmu-miR-148b-3p | MIMAT0000580 | 1.662999988 | 0.352999985 | 1.16900003 |
| rno-miR-207 | MIMAT0003115 | 1.631000042 | 1.427000046 | 1.327000022 |
| mmu-miR-24-1-5p | MIMAT0000218 | 1.61500001 | 6.896999836 | 1.00999999 |
| mmu-miR-539-5p | MIMAT0003169 | 1.603000045 | 0.550000012 | 0.361999989 |
| mmu-miR-2135 | MIMAT0011211 | 1.588000059 | 1.93599999 | 0.564999998 |
| mmu-miR-21-5p | MIMAT0000530 | 1.58099997 | 0.600000024 | 1.118000031 |
| mmu-miR-130a-3p | MIMAT0000141 | 1.557999969 | 0.611999989 | 1.167000055 |
| mmu-miR-200c-3p | MIMAT0000657 | 1.555999994 | 1.378999949 | 2.128999949 |
| mmu-let-7c-5p | MIMAT0000523 | 1.554999948 | 0.856000006 | 0.675000012 |
| mmu-miR-10a-5p | MIMAT0000648 | 1.552000046 | 1.123999953 | 0.795000017 |
| mmu-miR-199a-5p | MIMAT0000229 | 1.539000034 | 29.87199974 | 10.90200043 |
| rno-miR-532-5p | MIMAT0005322 | 1.523000002 | 1.93599999 | 0.869000018 |
| mmu-miR-320-3p | MIMAT0000666 | 1.506999969 | 0.555999994 | 1.717000008 |
| rno-miR-29c-5p | MIMAT0003154 | 0.697000027 | 1.230000019 | 0.860000014 |
| mmu-miR-138-5p | MIMAT0000150 | 0.694999993 | 0.545000017 | 1.194000006 |
| hsa-miR-421 | MIMAT0003339 | 0.694999993 | 0.713999987 | 0.749000013 |
| mmu-miR-351-5p | MIMAT0000609 | 0.690999985 | 0.920000017 | 0.515999973 |
| hsa-miR-93-3p | MIMAT0004509 | 0.68900001 | 1.085000038 | 0.625999987 |
| mmu-miR-128-3p | MIMAT0000140 | 0.68599999 | 0.354999989 | 0.740999997 |
| mmu-miR-34a-5p | MIMAT0000542 | 0.684000015 | 0.653999984 | 0.887000024 |
| mmu-miR-335-3p | MIMAT0004704 | 0.683000028 | 0.435000002 | 0.786000013 |
| mmu-miR-221-3p | MIMAT0000669 | 0.67900002 | 0.456 | 0.441000015 |
| hsa-miR-29a-5p | MIMAT0004503 | 0.676999986 | 0.63499999 | 1.694000006 |
| mmu-miR-133b-3p | MIMAT0000769 | 0.674000025 | 0.904999971 | 1.047000051 |
| hsa-miR-106b-3p | MIMAT0004672 | 0.670000017 | 0.842000008 | 2.680000067 |
| mmu-miR-151-3p | MIMAT0000161 | 0.663999975 | 0.870999992 | 1.315000057 |
| mmu-miR-19b-3p | MIMAT0000513 | 0.661000013 | 0.68900001 | 0.606000006 |
| mmu-miR-15a-5p | MIMAT0000526 | 0.640999973 | 0.43599999 | 0.552999973 |
| mmu-miR-15a-5p | MIMAT0000585 | 0.633000016 | 1.523000002 | 1.046000004 |
| mmu-miR-145a-5p | MIMAT0000157 | 0.629999995 | 0.561999977 | 0.791000009 |
| mmu-miR-200b-3p | MIMAT0000233 | 0.629999995 | 3.464999914 | 0.216999993 |
| mmu-miR-28a-3p | MIMAT0004661 | 0.629000008 | 0.873000026 | 0.214000002 |
| mmu-miR-135b-5p | MIMAT0000612 | 0.628000021 | 0.851000011 | 1.057000041 |
| mmu-miR-1839-5p | MIMAT0009456 | 0.624000013 | 1.083999991 | 1.054999948 |
| mmu-miR-19a-3p | MIMAT0000651 | 0.620999992 | 0.326999992 | 1.401000023 |
| mmu-miR-1198-5p | MIMAT0005859 | 0.619000018 | 0.432000011 | 1.118999958 |
| mmu-miR-674-5p | MIMAT0003740 | 0.616999984 | 0.648999989 | 0.681999981 |
| mmu-miR-376a-3p | MIMAT0000740 | 0.615999997 | 0.115000002 | 0.059 |
| mmu-miR-376c-3p | MIMAT0003183 | 0.61500001 | 0.32100001 | 0.231000006 |
| hsa-miR-190b-5p | MIMAT0004929 | 0.614000022 | 0.832000017 | 0.527999997 |
| mmu-miR-331-5p | MIMAT0004643 | 0.606999993 | 3.555000067 | 1.40199995 |
| hsa-miR-136-3p | MIMAT0004606 | 0.595000029 | 0.675999999 | 9.005000114 |
| mmu-miR-186-3p | MIMAT0004540 | 0.595000029 | 0.526000023 | 0.651000023 |
| mmu-miR-411-5p | MIMAT0004747 | 0.587000012 | 0.546000004 | 0.75999999 |
| rno-miR-7a-1-3p | MIMAT0000607 | 0.583000004 | 1.434000015 | 1.501000047 |
| mmu-miR-139-5p | MIMAT0000656 | 0.57099998 | 0.629000008 | 1.074000001 |
| mmu-miR-127-3p | MIMAT0000139 | 0.566999972 | 0.693000019 | 0.433999985 |
| mmu-miR-712-5p | MIMAT0003502 | 0.560000002 | 0.721000016 | 1.052000046 |
| mmu-miR-30d-5p | MIMAT0000515 | 0.555000007 | 0.888999999 | 0.609000027 |
| mmu-miR-143-3p | MIMAT0000247 | 0.552999973 | 0.575999975 | 0.727999985 |
| mmu-miR-30c-5p | MIMAT0000514 | 0.551999986 | 0.425999999 | 0.566999972 |
| hsa-miR-30e-3p | MIMAT0000693 | 0.551999986 | 0.845000029 | 0.737999976 |
| hsa-miR-455-5p | MIMAT0003150 | 0.551999986 | 0.626999974 | 0.721000016 |
| mmu-miR-136-5p | MIMAT0000148 | 0.550000012 | 0.166999996 | 1.238999963 |
| hsa-miR-494-3p | MIMAT0002816 | 0.546000004 | 0.264999986 | 0.363000005 |
| mmu-miR-451a | MIMAT0001632 | 0.54400003 | 0.595000029 | 0.874000013 |
| hsa-miR-30d-3p | MIMAT0004551 | 0.542999983 | 0.75999999 | 0.81400001 |
| hsa-miR-223-3p | MIMAT0000280 | 0.541000009 | 1.203999996 | 1.149000049 |
| mmu-miR-2146 | MIMAT0011222 | 0.537999988 | 0.535000026 | 0.610000014 |
| mmu-miR-126-5p | MIMAT0000137 | 0.533999979 | 0.240999997 | 1.037999988 |
| mmu-miR-673-5p | MIMAT0003739 | 0.532999992 | 0.328000009 | 0.414999992 |
| mmu-miR-187-3p | MIMAT0000216 | 0.529999971 | 1.442999959 | 0.358999997 |
| mmu-miR-1901 | MIMAT0007880 | 0.526000023 | 2.789000034 | 3.621999979 |
| rno-miR-20a-3p | MIMAT0000603 | 0.522000015 | 0.606000006 | 0.564999998 |
| mmu-let-7a-5p | MIMAT0000521 | 0.518999994 | 0.638999999 | 0.529999971 |
| mmu-miR-185-5p | MIMAT0000214 | 0.518000007 | 0.370000005 | 0.971000016 |
| mmu-miR-2182 | MIMAT0011286 | 0.518000007 | 3.056999922 | 0.54400003 |
| mmu-miR-503-5p | MIMAT0003188 | 0.515999973 | 0.453999996 | 0.351000011 |
| mmu-miR-7a-1-3p | MIMAT0004670 | 0.50999999 | 1.177999973 | 1.338000059 |
| mmu-miR-204-5p | MIMAT0000237 | 0.497000009 | 0.419 | 0.697000027 |
| mmu-miR-497a-5p | MIMAT0003453 | 0.493999988 | 0.744000018 | 1.360999942 |
| mmu-miR-431-5p | MIMAT0001418 | 0.485000014 | 1.317999959 | 0.101999998 |
| mmu-miR-361-5p | MIMAT0000704 | 0.477999985 | 0.059 | 0.711000025 |
| mmu-miR-467b-3p | MIMAT0003478 | 0.472000003 | 2.118000031 | 2.207999945 |
| mmu-miR-335-5p | MIMAT0000766 | 0.449000001 | 0.414000005 | 0.646000028 |
| mmu-miR-676-5p | MIMAT0003781 | 0.439999998 | 0.048 | 0.181999996 |
| hsa-miR-33a-3p | MIMAT0004506 | 0.42899999 | 0.241999999 | 0.630999982 |
| mmu-miR-592-5p | MIMAT0003730 | 0.428000003 | 0.224000007 | 0.377000004 |
| mmu-let-7a-1-3p | MIMAT0004620 | 0.412 | 0.824999988 | 0.230000004 |
| mmu-miR-100-5p | MIMAT0000655 | 0.411000013 | 0.449999988 | 0.690999985 |
| mmu-miR-1a-3p | MIMAT0000123 | 0.407999992 | 0.324000001 | 0.395999998 |
| mmu-miR-149-5p | MIMAT0000159 | 0.40200001 | 1.406999946 | 0.785000026 |
| mmu-miR-2134 | MIMAT0011210 | 0.393999994 | 0.834999979 | 0.453999996 |
| mmu-miR-425-3p | MIMAT0001342 | 0.39199999 | 1.345999956 | 0.426999986 |
| mmu-miR-133a-3p | MIMAT0000145 | 0.38499999 | 0.90200001 | 0.626999974 |
| mmu-miR-1937c | MIMAT0009429 | 0.379999995 | 0.202999994 | 0.314999998 |
| hsa-miR-299-5p | MIMAT0002890 | 0.375 | 0.171000004 | 0.312999994 |
| mmu-miR-1960 | MIMAT0009433 | 0.356999993 | 0.003 | 0.523999989 |
| mmu-miR-190a-5p | MIMAT0000220 | 0.345999986 | 0.980000019 | 0.609000027 |
| mmu-miR-706 | MIMAT0003496 | 0.338999987 | 0.680000007 | 0.31400001 |
| hsa-miR-378a-5p | MIMAT0000731 | 0.338 | 0.620999992 | 0.52700001 |
| mmu-miR-107-3p | MIMAT0000647 | 0.331 | 0.540000021 | 4.243000031 |
| mmu-miR-1981-5p | MIMAT0009458 | 0.324999988 | 1.292000055 | 0.83099997 |
| mmu-miR-376b-5p | MIMAT0003388 | 0.324000001 | 1.434000015 | 1.700000048 |
| mmu-miR-1928 | MIMAT0009391 | 0.32100001 | 0.558000028 | 1.764999986 |
| hsa-miR-206-3p | MIMAT0000462 | 0.316000015 | 0.72299999 | 4.106999874 |
| hsa-miR-214-5p | MIMAT0004564 | 0.310000002 | 0.521000028 | 0.31400001 |
| mmu-miR-135a-5p | MIMAT0000147 | 0.305999994 | 0.958999991 | 0.472000003 |
| hsa-miR-22-5p | MIMAT0004495 | 0.301999986 | 0.677999973 | 0.284000009 |
| mmu-miR-9-5p | MIMAT0000142 | 0.296999991 | 1.023000002 | 0.559000015 |
| rno-miR-101b-3p | MIMAT0000615 | 0.291999996 | 0.425999999 | 0.38499999 |
| mmu-miR-1943 | MIMAT0009408 | 0.287999988 | 0.542999983 | 0.660000026 |
| mmu-miR-338-3p | MIMAT0000582 | 0.279000014 | 0.014 | 0.039000001 |
| mmu-miR-297a-3p | MIMAT0004864 | 0.277999997 | 6.28399992 | 0.006 |
| rno-miR-673-5p | MIMAT0005328 | 0.272000015 | 1.10800004 | 0.564999998 |
| mmu-miR-700-3p | MIMAT0003490 | 0.272000015 | 0.263000011 | 0.611999989 |
| rno-miR-345-5p | MIMAT0000594 | 0.270000011 | 0.606999993 | 0.65200001 |
| hsa-miR-10b-5p | MIMAT0000254 | 0.261000007 | 0.730000019 | 0.079999998 |
| hsa-miR-744-3p | MIMAT0004946 | 0.246000007 | 0.712000012 | 0.513000011 |
| mmu-miR-1274a | MIMAT0009445 | 0.243000001 | 0.342999995 | 0.591000021 |
| mmu-miR-1937b | MIMAT0009414 | 0.237000003 | 0.398000002 | 0.416000009 |
| rno-miR-25-5p | MIMAT0004713 | 0.236000001 | 0.177000001 | 0.002 |
| mmu-miR-690 | MIMAT0003469 | 0.231999993 | 0.270999998 | 0.206 |
| mmu-miR-503-3p | MIMAT0004790 | 0.224000007 | 0.862999976 | 0.317000002 |
| mmu-miR-1903 | MIMAT0007868 | 0.211999997 | 0.057 | 0.328000009 |
| mmu-miR-380-3p | MIMAT0000745 | 0.209999993 | 1.682000041 | 3.740999937 |
| mmu-miR-322-3p | MIMAT0000549 | 0.209999993 | 0.476999998 | 1.638000011 |
| mmu-miR-1961 | MIMAT0009434 | 0.204999998 | 1.00999999 | 0.540000021 |
| rno-miR-99a-3p | MIMAT0004724 | 0.187999994 | 1.550999999 | 5.697000027 |
| mmu-miR-30b-3p | MIMAT0004524 | 0.160999998 | 1.235999942 | 0.012 |
| mmu-miR-455-3p | MIMAT0003742 | 0.152999997 | 0.244000003 | 0.014 |
| mmu-miR-542-5p | MIMAT0003171 | 0.138999999 | 1.014999986 | 2.588999987 |
| mmu-miR-1896 | MIMAT0007873 | 0.129999995 | 2.625 | 0.419999987 |
| mmu-miR-322 | MIMAT0000547 | 0.107000001 | 1.606999993 | 0.395000011 |
| mmu-miR-1954 | MIMAT0009425 | 0.093000002 | 0.88499999 | 0.194999993 |
| mmu-miR-672-5p | MIMAT0003735 | 0.079000004 | 0.02 | 0.01 |
| mmu-miR-509-3p | MIMAT0004891 | 0.077 | 0.012 | 0.897000015 |
| hsa-miR-9-3p | MIMAT0000442 | 0.068999998 | 0.725000024 | 0.381999999 |
| hsa-miR-338-5p | MIMAT0004701 | 0.046999998 | 0.225999996 | 0.149000004 |
| hsa-let-7f-1-3p | MIMAT0004486 | 0.041999999 | 0.497000009 | 0.218999997 |
| mmu-miR-708-5p | MIMAT0004828 | 0.037 | 0.342999995 | 0.001 |
| rno-miR-489-3p | MIMAT0003113 | 0.025 | 0.004 | 0.017999999 |
| hsa-miR-144-3p | MIMAT0000436 | 0.023 | 0.100000001 | 0.041000001 |
| mmu-miR-141-3p | MIMAT0000153 | 0.016000001 | 0.709999979 | 7.544000149 |
| mmu-miR-504-5p | MIMAT0004889 | 0.014 | 0.31099999 | 0.637000024 |
