# Supplementary material for: Treatment with benznidazole and pentoxifylline regulates microRNA transcriptomic profile in a murine model of Chagas chronic cardiomyopathy
Source: PLoS Negl Trop Dis. 2023 Mar 27;17(3):e0011223. doi: 10.1371/journal.pntd.0011223 (PMC10121046; doi:10.1371/journal.pntd.0011223)
Supplement: S2 Table — (DOCX) [file pntd.0011223.s002.docx]

**Supplementary table 2.** microRNAs restored to levels of uninfected mice (between 1.5-fold-change) after Bz treatment.

| **Name** | **Acession Number** | **Infected** | **Bz** | **Bz+PTX** |
| --- | --- | --- | --- | --- |
| mmu-miR-467b-5p | MIMAT0005448 | 8.119999886 | 1.129999995 | 5.899000168 |
| rno-miR-547-3p | MIMAT0012851 | 7.427000046 | 0.758000016 | 1.972000003 |
| mmu-miR-146b-5p | MIMAT0003475 | 4.070000172 | 1.396000028 | 1.222000003 |
| mmu-miR-466g | MIMAT0004883 | 3.582000017 | 1.424999952 | 1.521000028 |
| mmu-miR-210-3p | MIMAT0000658 | 3.394000053 | 1.368999958 | 1.06099999 |
| mmu-miR-669n | MIMAT0009427 | 3.295000076 | 0.757000029 | 1.205000043 |
| rno-miR-339-3p | MIMAT0004648 | 2.921000004 | 1.233999968 | 7.90199995 |
| mmu-miR-142-5p | MIMAT0000154 | 2.91899991 | 1.187000036 | 0.30399999 |
| mmu-miR-467d-3p | MIMAT0004887 | 2.760999918 | 1.050999999 | 1.309999943 |
| mmu-miR-29b-3p | MIMAT0000127 | 2.569000006 | 1.120000005 | 0.939999998 |
| hsa-miR-200b-5p | MIMAT0004571 | 2.536999941 | 1.011000037 | 1.440999985 |
| mmu-miR-342-3p | MIMAT0000590 | 2.430000067 | 1.013000011 | 1.980999947 |
| mmu-miR-2183 | MIMAT0011287 | 2.323999882 | 1.324000001 | 3.499000072 |
| mmu-miR-694 | MIMAT0003474 | 2.227999926 | 1.110999942 | 0.563000023 |
| mmu-miR-324-5p | MIMAT0000555 | 2.174000025 | 1.353000045 | 1.210999966 |
| mmu-miR-547-3p | MIMAT0003173 | 2.049000025 | 0.734000027 | 1.149000049 |
| rno-miR-146b-5p | MIMAT0005595 | 1.967000008 | 1.279000044 | 1.126999974 |
| mmu-miR-132-3p | MIMAT0000144 | 1.860000014 | 1.213000059 | 0.948000014 |
| mmu-miR-1188-5p | MIMAT0005843 | 1.792000055 | 1.348999977 | 2.503999949 |
| mmu-miR-223-3p | MIMAT0000665 | 1.784999967 | 1.167000055 | 1.583999991 |
| mmu-miR-296-5p | MIMAT0000374 | 1.741999984 | 1.43599999 | 1.465999961 |
| mmu-miR-339-3p | MIMAT0004649 | 1.72300005 | 1.144999981 | 2.039000034 |
| rno-miR-207 | MIMAT0003115 | 1.631000042 | 1.427000046 | 1.327000022 |
| mmu-miR-200c-3p | MIMAT0000657 | 1.555999994 | 1.378999949 | 2.128999949 |
| mmu-let-7c-5p | MIMAT0000523 | 1.554999948 | 0.856000006 | 0.675000012 |
| mmu-miR-10a-5p | MIMAT0000648 | 1.552000046 | 1.123999953 | 0.795000017 |
| rno-miR-29c-5p | MIMAT0003154 | 0.697000027 | 1.230000019 | 0.860000014 |
| hsa-miR-421 | MIMAT0003339 | 0.694999993 | 0.713999987 | 0.749000013 |
| mmu-miR-351-5p | MIMAT0000609 | 0.690999985 | 0.920000017 | 0.515999973 |
| hsa-miR-93-3p | MIMAT0004509 | 0.68900001 | 1.085000038 | 0.625999987 |
| mmu-miR-133b-3p | MIMAT0000769 | 0.674000025 | 0.904999971 | 1.047000051 |
| hsa-miR-106b-3p | MIMAT0004672 | 0.670000017 | 0.842000008 | 2.680000067 |
| mmu-miR-151-3p | MIMAT0000161 | 0.663999975 | 0.870999992 | 1.315000057 |
| mmu-miR-28a-3p | MIMAT0004661 | 0.629000008 | 0.873000026 | 0.214000002 |
| mmu-miR-135b-5p | MIMAT0000612 | 0.628000021 | 0.851000011 | 1.057000041 |
| mmu-miR-1839-5p | MIMAT0009456 | 0.624000013 | 1.083999991 | 1.054999948 |
| hsa-miR-190b-5p | MIMAT0004929 | 0.614000022 | 0.832000017 | 0.527999997 |
| rno-miR-7a-1-3p | MIMAT0000607 | 0.583000004 | 1.434000015 | 1.501000047 |
| mmu-miR-712-5p | MIMAT0003502 | 0.560000002 | 0.721000016 | 1.052000046 |
| mmu-miR-30d-5p | MIMAT0000515 | 0.555000007 | 0.888999999 | 0.609000027 |
| hsa-miR-30e-3p | MIMAT0000693 | 0.551999986 | 0.845000029 | 0.737999976 |
| hsa-miR-30d-3p | MIMAT0004551 | 0.542999983 | 0.75999999 | 0.81400001 |
| hsa-miR-223-3p | MIMAT0000280 | 0.541000009 | 1.203999996 | 1.149000049 |
| mmu-miR-187-3p | MIMAT0000216 | 0.529999971 | 1.442999959 | 0.358999997 |
| mmu-miR-7a-1-3p | MIMAT0004670 | 0.50999999 | 1.177999973 | 1.338000059 |
| mmu-miR-497a-5p | MIMAT0003453 | 0.493999988 | 0.744000018 | 1.360999942 |
| mmu-miR-431-5p | MIMAT0001418 | 0.485000014 | 1.317999959 | 0.101999998 |
| mmu-let-7a-1-3p | MIMAT0004620 | 0.412 | 0.824999988 | 0.230000004 |
| mmu-miR-149-5p | MIMAT0000159 | 0.40200001 | 1.406999946 | 0.785000026 |
| mmu-miR-2134 | MIMAT0011210 | 0.393999994 | 0.834999979 | 0.453999996 |
| mmu-miR-425-3p | MIMAT0001342 | 0.39199999 | 1.345999956 | 0.426999986 |
| mmu-miR-133a-3p | MIMAT0000145 | 0.38499999 | 0.90200001 | 0.626999974 |
| mmu-miR-190a-5p | MIMAT0000220 | 0.345999986 | 0.980000019 | 0.609000027 |
| mmu-miR-1981-5p | MIMAT0009458 | 0.324999988 | 1.292000055 | 0.83099997 |
| mmu-miR-376b-5p | MIMAT0003388 | 0.324000001 | 1.434000015 | 1.700000048 |
| hsa-miR-206-3p | MIMAT0000462 | 0.316000015 | 0.72299999 | 4.106999874 |
| mmu-miR-135a-5p | MIMAT0000147 | 0.305999994 | 0.958999991 | 0.472000003 |
| mmu-miR-9-5p | MIMAT0000142 | 0.296999991 | 1.023000002 | 0.559000015 |
| rno-miR-673-5p | MIMAT0005328 | 0.272000015 | 1.10800004 | 0.564999998 |
| hsa-miR-10b-5p | MIMAT0000254 | 0.261000007 | 0.730000019 | 0.079999998 |
| hsa-miR-744-3p | MIMAT0004946 | 0.246000007 | 0.712000012 | 0.513000011 |
| mmu-miR-503-3p | MIMAT0004790 | 0.224000007 | 0.862999976 | 0.317000002 |
| mmu-miR-1961 | MIMAT0009434 | 0.204999998 | 1.00999999 | 0.540000021 |
| mmu-miR-30b-3p | MIMAT0004524 | 0.160999998 | 1.235999942 | 0.012 |
| mmu-miR-542-5p | MIMAT0003171 | 0.138999999 | 1.014999986 | 2.588999987 |
| mmu-miR-1954 | MIMAT0009425 | 0.093000002 | 0.88499999 | 0.194999993 |
| hsa-miR-9-3p | MIMAT0000442 | 0.068999998 | 0.725000024 | 0.381999999 |
| mmu-miR-141-3p | MIMAT0000153 | 0.016000001 | 0.709999979 | 7.544000149 |
