# Supplementary material for: Treatment with benznidazole and pentoxifylline regulates microRNA transcriptomic profile in a murine model of Chagas chronic cardiomyopathy
Source: PLoS Negl Trop Dis. 2023 Mar 27;17(3):e0011223. doi: 10.1371/journal.pntd.0011223 (PMC10121046; doi:10.1371/journal.pntd.0011223)
Supplement: S3 Table — (DOCX) [file pntd.0011223.s003.docx]

**Supplementary table 3.** microRNAs restored to levels of uninfected mice (between 1.5-fold-change) after Bz+PTX treatment.

| **Name** | **Acession Number** | **Infected** | **Bz** | **Bz+PTX** |
| --- | --- | --- | --- | --- |
| mmu-miR-878-3p | MIMAT0004933 | 127.887001 | 3.227999926 | 1.351999998 |
| rno-miR-196c-5p | MIMAT0005303 | 10.1260004 | 0.135000005 | 0.773999989 |
| mmu-miR-146b-5p | MIMAT0003475 | 4.070000172 | 1.396000028 | 1.222000003 |
| mmu-miR-1930 | MIMAT0009393 | 3.460000038 | 1.501000047 | 1.207000017 |
| mmu-miR-210-3p | MIMAT0000658 | 3.394000053 | 1.368999958 | 1.06099999 |
| mmu-miR-669n | MIMAT0009427 | 3.295000076 | 0.757000029 | 1.205000043 |
| mmu-miR-467d-3p | MIMAT0004887 | 2.760999918 | 1.050999999 | 1.309999943 |
| mmu-miR-29b-3p | MIMAT0000127 | 2.569000006 | 1.120000005 | 0.939999998 |
| hsa-miR-200b-5p | MIMAT0004571 | 2.536999941 | 1.011000037 | 1.440999985 |
| mmu-miR-324-5p | MIMAT0000555 | 2.174000025 | 1.353000045 | 1.210999966 |
| mmu-miR-490-3p | MIMAT0003780 | 2.072999954 | 2.094000101 | 1.457000017 |
| mmu-miR-547-3p | MIMAT0003173 | 2.049000025 | 0.734000027 | 1.149000049 |
| rno-miR-351-5p | MIMAT0000608 | 2.025000095 | 0.463999987 | 1.45599997 |
| rno-miR-350 | MIMAT0000604 | 1.976999998 | 4.551000118 | 1.11500001 |
| rno-miR-146b-5p | MIMAT0005595 | 1.967000008 | 1.279000044 | 1.126999974 |
| mmu-miR-34b-3p | MIMAT0004581 | 1.919999957 | 0.68900001 | 0.716000021 |
| mmu-miR-132-3p | MIMAT0000144 | 1.860000014 | 1.213000059 | 0.948000014 |
| mmu-miR-296-5p | MIMAT0000374 | 1.741999984 | 1.43599999 | 1.465999961 |
| mmu-miR-148b-3p | MIMAT0000580 | 1.662999988 | 0.352999985 | 1.16900003 |
| rno-miR-207 | MIMAT0003115 | 1.631000042 | 1.427000046 | 1.327000022 |
| mmu-miR-24-1-5p | MIMAT0000218 | 1.61500001 | 6.896999836 | 1.00999999 |
| mmu-miR-21-5p | MIMAT0000530 | 1.58099997 | 0.600000024 | 1.118000031 |
| mmu-miR-130a-3p | MIMAT0000141 | 1.557999969 | 0.611999989 | 1.167000055 |
| mmu-miR-10a-5p | MIMAT0000648 | 1.552000046 | 1.123999953 | 0.795000017 |
| rno-miR-532-5p | MIMAT0005322 | 1.523000002 | 1.93599999 | 0.869000018 |
| rno-miR-29c-5p | MIMAT0003154 | 0.697000027 | 1.230000019 | 0.860000014 |
| mmu-miR-138-5p | MIMAT0000150 | 0.694999993 | 0.545000017 | 1.194000006 |
| hsa-miR-421 | MIMAT0003339 | 0.694999993 | 0.713999987 | 0.749000013 |
| mmu-miR-128-3p | MIMAT0000140 | 0.68599999 | 0.354999989 | 0.740999997 |
| mmu-miR-34a-5p | MIMAT0000542 | 0.684000015 | 0.653999984 | 0.887000024 |
| mmu-miR-335-3p | MIMAT0004704 | 0.683000028 | 0.435000002 | 0.786000013 |
| mmu-miR-133b-3p | MIMAT0000769 | 0.674000025 | 0.904999971 | 1.047000051 |
| mmu-miR-151-3p | MIMAT0000161 | 0.663999975 | 0.870999992 | 1.315000057 |
| mmu-miR-15a-5p | MIMAT0000585 | 0.633000016 | 1.523000002 | 1.046000004 |
| mmu-miR-145a-5p | MIMAT0000157 | 0.629999995 | 0.561999977 | 0.791000009 |
| mmu-miR-135b-5p | MIMAT0000612 | 0.628000021 | 0.851000011 | 1.057000041 |
| mmu-miR-1839-5p | MIMAT0009456 | 0.624000013 | 1.083999991 | 1.054999948 |
| mmu-miR-19a-3p | MIMAT0000651 | 0.620999992 | 0.326999992 | 1.401000023 |
| mmu-miR-1198-5p | MIMAT0005859 | 0.619000018 | 0.432000011 | 1.118999958 |
| mmu-miR-331-5p | MIMAT0004643 | 0.606999993 | 3.555000067 | 1.40199995 |
| mmu-miR-411-5p | MIMAT0004747 | 0.587000012 | 0.546000004 | 0.75999999 |
| mmu-miR-139-5p | MIMAT0000656 | 0.57099998 | 0.629000008 | 1.074000001 |
| mmu-miR-712-5p | MIMAT0003502 | 0.560000002 | 0.721000016 | 1.052000046 |
| mmu-miR-143-3p | MIMAT0000247 | 0.552999973 | 0.575999975 | 0.727999985 |
| hsa-miR-30e-3p | MIMAT0000693 | 0.551999986 | 0.845000029 | 0.737999976 |
| hsa-miR-455-5p | MIMAT0003150 | 0.551999986 | 0.626999974 | 0.721000016 |
| mmu-miR-136-5p | MIMAT0000148 | 0.550000012 | 0.166999996 | 1.238999963 |
| mmu-miR-451a | MIMAT0001632 | 0.54400003 | 0.595000029 | 0.874000013 |
| hsa-miR-30d-3p | MIMAT0004551 | 0.542999983 | 0.75999999 | 0.81400001 |
| hsa-miR-223-3p | MIMAT0000280 | 0.541000009 | 1.203999996 | 1.149000049 |
| mmu-miR-126-5p | MIMAT0000137 | 0.533999979 | 0.240999997 | 1.037999988 |
| mmu-miR-185-5p | MIMAT0000214 | 0.518000007 | 0.370000005 | 0.971000016 |
| mmu-miR-7a-1-3p | MIMAT0004670 | 0.50999999 | 1.177999973 | 1.338000059 |
| mmu-miR-497a-5p | MIMAT0003453 | 0.493999988 | 0.744000018 | 1.360999942 |
| mmu-miR-361-5p | MIMAT0000704 | 0.477999985 | 0.059 | 0.711000025 |
| mmu-miR-149-5p | MIMAT0000159 | 0.40200001 | 1.406999946 | 0.785000026 |
| mmu-miR-1981-5p | MIMAT0009458 | 0.324999988 | 1.292000055 | 0.83099997 |
| mmu-miR-509-3p | MIMAT0004891 | 0.077 | 0.012 | 0.897000015 |
